# Physiological and transcriptional analyses of Arabidopsis primary root growth in response to phosphate starvation under light and dark conditions

**DOI:** 10.3389/fpls.2025.1557118

**Published:** 2025-04-10

**Authors:** Zhen Wang, Mingzhe Xia, Rui Ma, Zai Zheng

**Affiliations:** ^1^ School of Agriculture, Forestry and Medicine, The Open University of China, Beijing, China; ^2^ State Key Laboratory of Plateau Ecology and Agriculture, Qinghai University, Xining, China; ^3^ Ministry of Education Key Laboratory of Bioinformatics, Center for Plant Biology, School of Life Sciences, Tsinghua University, Beijing, China; ^4^ National Key Laboratory of Tropical Crop Breeding, Institute of Tropical Bioscience and Biotechnology and Sanya Research Institute, Chinese Academy of Tropical Agricultural Sciences, Hainan, China

**Keywords:** Pi starvation, light illumination, primary root growth, transcriptomic analyses, cryptochromes, HY5

## Abstract

Plants cope with Pi deficiency by triggering an array of adaptive responses, including the remodeling of root system architecture (RSA). *Arabidopsis thaliana* grown on a Pi-deficient (-Pi) medium in transparent Petri dishes exhibits an inhibition of primary root (PR) growth. Previous work has shown that direct illumination on roots by blue light is both required and sufficient for the Pi deficiency-induced inhibition of PR growth. However, whether light illumination on shoots of seedlings contributes to the inhibition of PR growth under -Pi condition and whether light signaling pathway is involved in this process remain largely unknown. In addition to Pi deficiency-induced inhibition of PR growth, how light affects the transcriptomic changes under -Pi also remains elusive. Here, we found that the inhibition of PR growth under -Pi condition is determined by light illumination on roots instead of shoots. Further experiments revealed that blue light receptors CRY1/CRY2 and key regulator in blue light signaling pathway HY5 play minor roles in this process. Finally, we evaluated the light effects on the transcriptomic changes during the inhibition of PR growth under -Pi condition. We found that light promotes the expression of many genes involved in stress and phytohormones-related processes and has both upregulated and downregulated effects on the expression of typical phosphate starvation-induced (PSI) genes. Taken together, our work further demonstrates our previous hypothesis that the inhibition of PR growth under -Pi condition is caused by blue light-triggered chemical reactions, rather than blue light signaling pathways. Apart from the inhibition of PR growth under -Pi, light exposure also results in substantial alterations of transcriptome under -Pi condition, encouraging us to carefully evaluate the phenotype under illuminated, transparent Petri dishes.

## Introduction

Phosphorus (P) is an essential macronutrient for plant growth, development, and metabolism. Plants uptake P from soil in the form of inorganic phosphate (Pi), which is quite limited in soils due to its low diffusion rate, fixation with metals, and conversion to organophosphates by microorganisms (Nussaume et al., 2011; Cong et al., 2020). To cope with Pi deficiency, plants have evolved sophisticated adaptive responses called Pi starvation responses (PSR) to sustain their growth, development, and reproduction (López-Arredondo et al., 2014). The major PSR of several plant species is the remodeling of root system architecture (RSA), including the inhibition of primary root (PR) growth, the increase of lateral roots and root hairs (Liu, 2021).

In the past decades, most studies of Arabidopsis root biology have used illuminated, transparent Petri dishes because it is simply easier to control the environment and collect data with experiments in Petri dishes than those in soil. When grown in Petri dishes under -Pi condition, Arabidopsis exhibits an inhibition of PR growth (Liu, 2021). Previous studies demonstrated that iron (Fe) accumulation in root apoplasts and secretion of malate into the rhizosphere are critical for Pi deficiency-induced inhibition of PR growth in Arabidopsis. Functional disruption of *LPR1* (a ferroxidase that oxidizes Fe^2+^ to Fe^3+^) and its close homolog *LPR2*, *ALMT1* (an aluminum-activated malate transporter), and *STOP1* (a transcription factor that directly regulates the transcription of *ALMT1*) reduced the accumulation of Fe^3+^ in root apoplasts and make plants insensitive to Pi deficiency-induced inhibition of PR growth (Svistoonoff et al., 2007; Müller et al., 2015; Balzergue et al., 2017; Mora-Macías et al., 2017; Wang et al., 2019). In contrast, mutation of *ALS3* (a transmembrane domain of a putative ABC transporter complex), *STAR1* (a nucleotide binding domain of a putative ABC transporter complex), two of the uncharacterized cytochrome b561 and DOMON domain (CYBDOM) protein family members, CRR and HYP1, over-accumulates Fe^3+^ in the root apoplasts and make plants hypersensitive to Pi deficiency-induced inhibition of PR growth (Dong et al., 2017; Clúa et al., 2024; Maniero et al., 2024).

Our laboratory previously reported that Pi deficiency-induced inhibition of PR growth is caused by a blue light-mediated Photo-Fenton reaction that couples with a canonical Fenton reaction to form a Fe redox cycle. The resultant Fe redox cycle generates hydroxyl radicals (·OH, a strong reactive oxygen species), thus inhibiting PR growth (Zheng et al., 2019). Although the strong inhibition of PR growth under -Pi condition is largely rescued by shielding of roots from light, which was supported by other research groups (Silva-Navas et al., 2019; Yeh et al., 2020; Gao H, et al., 2021; Tian et al., 2021), whether Pi deficiency-induced accumulations of Fe, callose, lignin, and ·OH are influenced by light illumination and the effects of light on transcriptomic changes under -Pi condition in roots have not been investigated. In contrast, Gao Y. Q. et al. (2021) claimed that inhibition of root elongation by -Pi requires blue light signal perception at the shoot and transduction to the root. In this process, after light perception by blue light receptor CRY1/CRY2, a crucial downstream component, HY5, migrates from shoot to root. The shoot-derived HY5 autoactivates root HY5 and regulates PR growth by directly activating the expression of *LPR1*. Another study revealed that the insensitive phenotype to PR growth inhibition of *hy5* mutant might be due to blockage of blue light responses (Yeh et al., 2020). Therefore, light exposure on which part (shoots or roots) is vital to trigger Pi deficiency-induced inhibition of PR growth and whether blue light signaling pathway contributes to this process remain to be investigated.

In the current study, we demonstrate that the inhibition of PR growth under -Pi condition is determined by light illumination on roots but not shoots. Further results revealed that blue light signaling pathway plays a minor role in this process. Through transcriptomic analyses, we found that light promotes the expression of a group of stress and phytohormone-related genes and has both upregulation and downregulation of the expression of typical PSI genes. These results support our previous hypothesis that the inhibition of PR growth under -Pi condition is caused by blue light-triggered chemical reactions. We therefore suggest that re-evaluation is needed of the many published studies that used illuminated Petri dishes to investigate root responses to environmental conditions.

## Materials and methods

### Plant materials and growth conditions

All Arabidopsis (*Arabidopsis thaliana*) plants used in this study were in the Columbia-0 background. The *cry1-104cry2-1* mutant (Mao et al., 2005) was kindly provided by Dr. Hongquan Yang (Shanghai Normal University, China). The *hy5-215* (Oyama et al., 1997), *HY5 OX-1*, and *HY5 OX-2* lines were kind gifts from Dr. Haodong Chen (Tsinghua University, China). The *lpr1lpr2* mutant (Svistoonoff et al., 2007) was kindly provided by Dr. Thierry Desnos (CEA Cadarache, France). The *LPR1 OX-1* and *LPR1 OX-2* lines were generated in our laboratory (Wang et al., 2019).

The Arabidopsis seeds were surface sterilized for 10 min with 20% (v/v) bleach and then washed three times with sterile-distilled water (ddH_2_O). After stratification at 4°C for 2 days, the seeds were sown on Petri dishes containing Pi-sufficient (+Pi) medium or Pi-deficient (-Pi) medium. The standard +Pi medium was half-strength Murashige and Skoog medium (Caisson Labs, MSP01-01190008) with 1% (w/v) sucrose, 0.1% (w/v) MES, and 0.8% (w/v) agarose (Biowest Regular Agarose G-10). For the -Pi medium, half-strength Murashige & Skoog without Pi (Caisson Labs, MSP11-05160009) was used to replace Murashige & Skoog basal salts. The pH was adjusted to 5.8 for both +Pi and -Pi media. The Petri dishes with Arabidopsis seeds were placed vertically in a phytotron with a photoperiod of 16 h of white light and 8 h of dark at 22-24°C. The intensity of white light was 100 µmol m^–2^ s^–1^.

To keep the shoots and roots in darkness, respectively, in light shielding experiment, we used autoclaved aluminum foil that was placed above the medium to cover the shoots or roots of 4-day-old seedlings. Moreover, we employed black plastic film in the rear of the plates to prevent the light from passing through the back (Zheng et al., 2019). The light was shone on the seedlings from above. Despite we did not insert the aluminum foil into the medium, as the transplanted 4-day-old seedlings were grown at a distance from the edges of the plates, the influence of light leakage from the sides that were not covered with aluminum foil on the root phenotypes might be negligible.

### Perls and Perls/DAB staining assays

The Perls staining method was performed as described with minor modifications (Roschzttardtz et al., 2009). Briefly, roots were stained with a Perls stain kit (Solarbio) for 30 min and rinsed with ddH_2_O twice. Then the stained roots were stored in 0.1 M Na-Phosphate buffer (pH 7.4).

The Perls/DAB staining was performed as described with small modifications (Balzergue et al., 2017). The final concentration of DAB solution was 0.025%. The stained roots were stored in 0.1 M Na-Phosphate buffer (pH 7.4). All stained samples were cleared on glass slides by 80% HCG clearing solution (diluted with 0.1 M Na-Phosphate buffer, pH 7.4). The Fe staining patterns in roots were examined using a 20x objective with a differential interference contrast (DIC) microscope (Olympus BX51) equipped with a camera (Olympus DP71).

### Callose staining assay

The callose staining was performed as described with minor modifications (Müller et al., 2015). Callose was stained with 0.1% (w/v) aniline blue (AppliChem) in 100 mM Na-phosphate buffer (pH 9.0) for 1.5 h. The callose staining patterns in roots were examined with a LSM710 confocal microscope (Zeiss).

### Lignin staining assay

The lignin staining was conducted as described with minor modifications (Ziegler et al., 2016). The roots were totally covered with 5% phloroglucinol solution and inoculated at room temperature for 3 min. The same volume of HCl solution was then added for 3 min in room temperature. Approximate 40% glycerol was added to the stained roots before observation with the microscope (Olympus BX51).

### Histochemical detection of ·OH in roots

The ·OH staining was conducted as described before (Setsukinai et al., 2003; Zheng et al., 2019). Roots were excised from 5-day-old seedlings and were pre-incubated in a 100 mM phosphate buffer (pH 6.1) for 20 min. The samples were then transferred to the same buffer containing 10 µM HPF (hydroxyphenyl fluorescein), and incubated for another 20 min. The fluorescent signals representing ·OH were observed with an LSM710 confocal microscope (Zeiss).

### Analysis of root-associated APase activity

For histochemical staining of APase activity on the root surface of Arabidopsis seedlings, an agar solution (0.5%, w/v) containing 0.01% (w/v) BCIP was evenly overlaid on the roots grown on agar plates (Wang et al., 2011). After 12 h of color development, the roots were photographed with a camera attached to a stereomicroscope (Olympus SZ61).

### Reverse transcription quantitative PCR analyses of PSI gene expression

Four-day-old +Pi seedlings were transferred to +Pi or -Pi medium with roots under light or in darkness for another four days. Then total RNA was extracted from roots using the Highpure Total RNA Mini kit (Magen). M-MLV reverse transcriptase (Takara) was used to reversely transcribe RNAs into cDNAs. SYRB Fast qPCR Master Mix (KAPA) was used for RT-qPCR analyses on a Bio-Rad CFX96 real-time PCR system. *ACTIN2* was used as an internal control, and the relative expression level of each gene was calculated by the 2^-ΔΔCt^ method (Livak and Schmittgen, 2001). The primers used for RT-qPCR analyses are listed in **Supplementary Table S1**.

### Transcriptomic analysis

Four-day-old +Pi seedlings were transferred to +Pi or -Pi medium with roots under light or in darkness for another four days. The total RNA was extracted from roots and 3 μg of RNA for each sample was used for library construction and subsequent RNA-deep sequencing on the Illumina Hiseq 2500 platform. Three biological replicates were used in this experiment. The adaptor sequences and low quality sequences were removed. Approximately 6.0 GB of clean reads were generated from each sample. The clean reads were mapped to the Arabidopsis reference genome (TAIR10) using TOPHAT v.2.1.0 with TAIR10 gene annotation as the transcript index. The minimum and maximum intron lengths were set to 40 and 5000 separately. CUFFLINKS v.2.2.1 was used to assemble the new transcripts. HTSEQ v.0.6.0 was used to calculate the raw read counts for each gene. Gene expression normalization among samples was performed by using DESEQ2. The different gene expression data were collected from the comparison with a fold change ≥ 2 and a false discovery rate (FDR, Benjamini-Hochberg adjusted P value) ≤ 0.01. Venn diagram was drawn by Venny 2.1.0 (https://bioinfogp.cnb.csic.es/tools/venny/). GO enrichment was performed using Metascape (https://metascape.org/gp/index.html). Heatmap analyses and hierarchical clustering were conducted by Morpheus (https://software.broadinstitute.org/morpheus/).

## Results

### Light illumination on roots, but not shoots, is both required and sufficient for Pi deficiency-induced inhibition of PR growth

**Figure 1:**
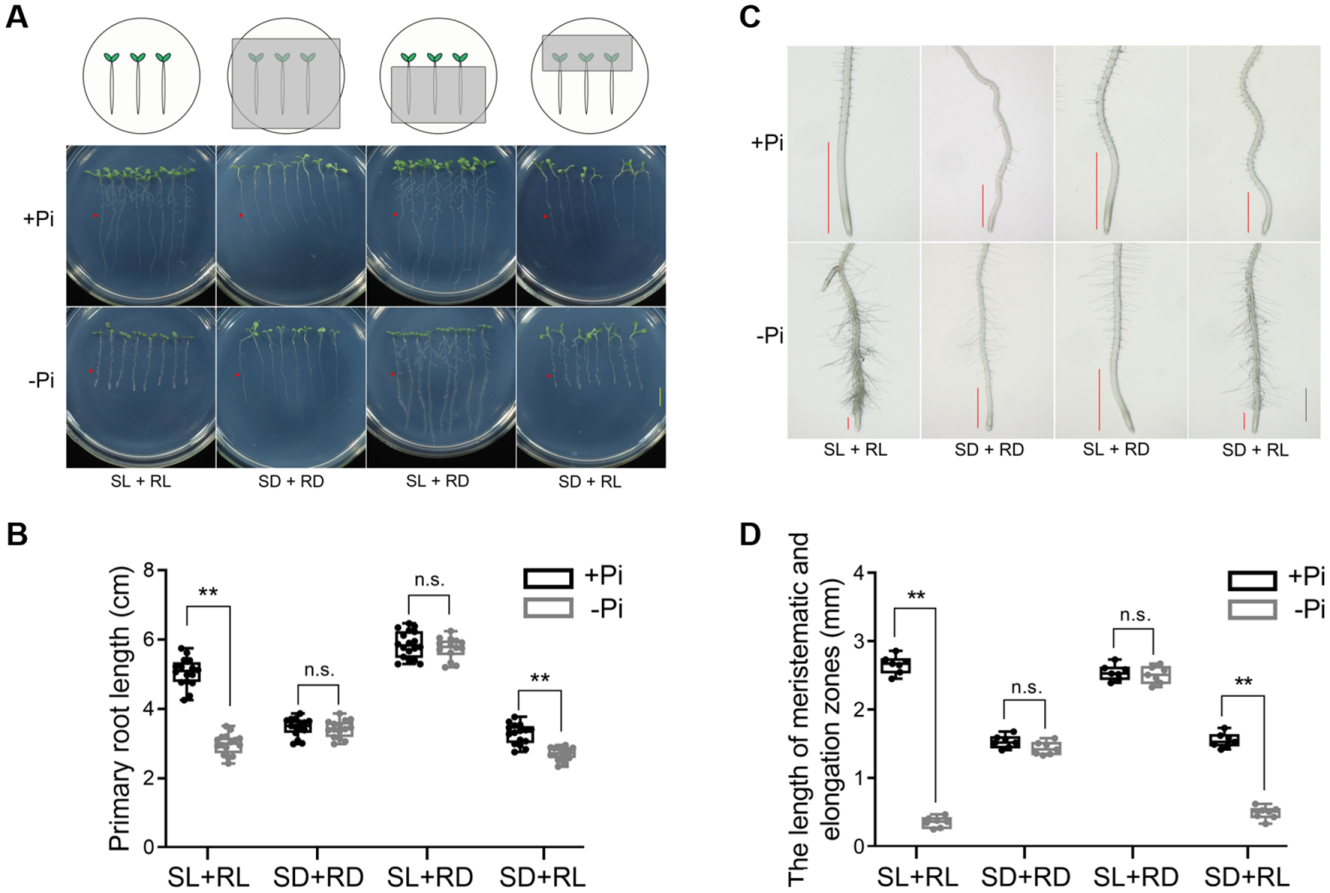
Light illumination on roots but not shoots is both required and sufficient for Pi deficiency-induced inhibition of PR growth. **(A)** Four-day-old +Pi WT seedlings were transferred to +Pi or –Pi medium and grown for another four days with shoots and roots exposed to different light conditions. SL: shoot under light; RL: root under light; SD: shoot in darkness. RD: root in darkness. The schematic diagrams on the top indicate how shoots and roots of the seedling were shielded from light by aluminum foil. The red triangles indicate the position of the root tips before the seedlings were transferred. The scale bar = 1 cm. **(B)** Quantification of the primary root length of the seedlings in **(A)**. The experiments were repeated three times, and the representative results are shown (median ± interquartile; Tukey whiskers; *n* = more than 10 seedlings per condition; **p < 0.01 in Student’s *t*-tests, n.s. represents no significant difference). **(C)** Morphologies of the root tips of the seedlings shown in **(A)**. The red lines indicate the size of meristematic and elongation zones. The scale bar = 1 mm. **(D)** Quantification of the length of meristematic and elongation zones in **(C)**. The experiments were repeated three times, and the representative results are shown (median ± interquartile; Tukey whiskers; *n* = 7 seedlings per condition; **p < 0.01 in Student’s *t*-tests, n.s. represents no significant difference).

Previous studies have revealed that Pi deficiency-induced inhibition of PR growth was largely rescued by shielding of roots from light (Silva-Navas et al., 2019; Zheng et al., 2019; Gao H, et al., 2021; Tian et al., 2021). To further assess the contributions of shoots and roots in Pi deficiency-induced inhibition of PR growth, we conducted a detailed light shielding experiment. The wild-type (WT) Arabidopsis seeds were directly germinated on Pi-sufficient (+Pi) medium. Four days after germination (DAG), the seedlings were transferred to +Pi or Pi-deficient (-Pi) medium under white light illumination with different shielding of shoots and roots (**Figure 1A**). At four days after transfer (DAT), the seedlings grown on -Pi medium with both shoots and roots exposed to white light (SL+RL) displayed the inhibition of PR growth compared to those grown on +Pi medium (**Figures 1A, B**). The root tips of these seedlings exhibited typical Pi deficiency-induced morphological changes, including the reduced meristem size, the increased root hair density and length, and the thickened cell walls (**Figures 1C, D**). For the seedlings with both shoots and roots grown in darkness (SD+RD), their PR growth was substantially reduced on both +Pi and -Pi media compared to SL+RL seedlings grown under +Pi condition (**Figures 1A, B**). Although the SD+RD seedlings showed reduced PR growth under -Pi condition, their root tips did not exhibit the Pi deficiency-induced morphological changes (**Figures 1C, D**).

To further distinguish the functions of shoots and roots in Pi deficiency-induced inhibition of PR growth, we then excluded shoots and roots from light, respectively, by shielding them with aluminum foil. Four-day-old seedlings grown +Pi medium were transferred to +Pi or -Pi medium with either shoots or roots shielded from light. At 4 DAT, the seedlings whose shoots were under light and roots were in darkness (SL+RD) no longer showed the inhibition of PR growth on -Pi medium (**Figures 1A, B**). The morphological changes in the root tips of these seedlings largely resembled those of SL+RL seedlings grown on +Pi medium (**Figures 1C, D**). In contrast, seedlings whose shoots were in the dark and roots were under light (SD+RL) exhibited retarded PR growth on +Pi medium compared with +Pi seedlings under SL+RL condition, and displayed further reduction on -Pi medium (**Figures 1A, B**). Unlike SD+RD seedlings, their root tips exhibited typical Pi deficiency-induced morphological changes, especially the reduced meristem size, on -Pi medium (**Figures 1C, D**). These results clearly demonstrated that the direct illumination on roots, instead of shoots, was both required and sufficient to mediate Pi deficiency-induced inhibition of PR growth.

### The Pi deficiency-induced accumulations of Fe, callose, lignin, and ·OH are attenuated in dark-grown roots

**Figure 2:**
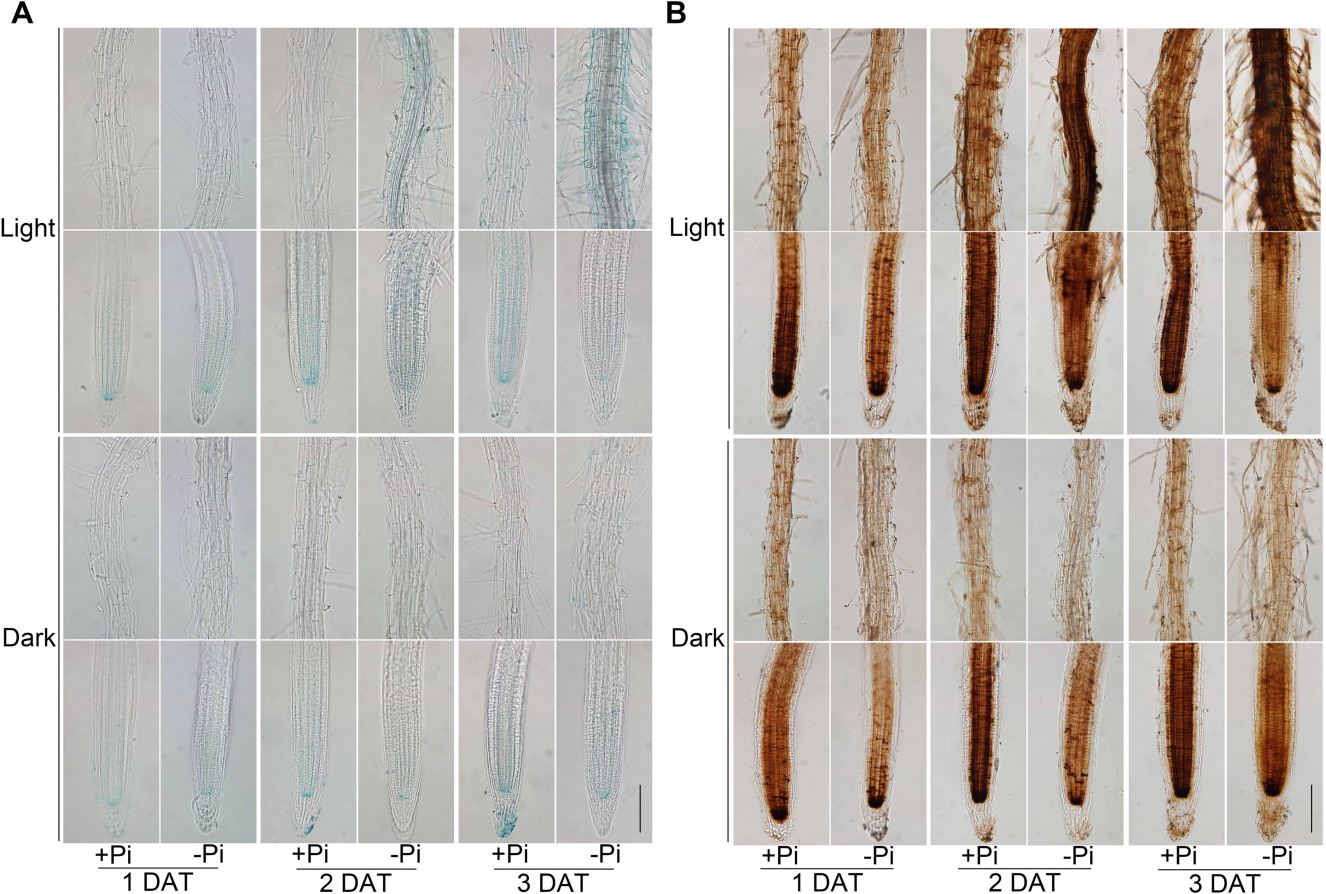
Iron staining patterns of the roots on +Pi and –Pi media under light or in darkness. The seeds were directly germinated on +Pi medium and grown for four days before they were transferred to +Pi and -Pi media under light or in darkness. Perls staining **(A)** and Perls/DAB staining **(B)** assays were performed at 1, 2, and 3 days after transfer (DAT). The upper and lower rows in each treatment are photographs of a part of the maturation zone and the root tip, respectively. The scale bar = 100 μm.

Previously, accumulation of Fe in root apoplasts has been shown to be crucial for Pi deficiency-induced inhibition of PR growth (Svistoonoff et al., 2007; Müller et al., 2015). We wondered whether the Fe accumulation under -Pi condition decreased when roots of seedlings were grown in darkness. We then examined the accumulation of Fe^3+^ and total Fe (Fe^2+^ and Fe^3+^ included) in roots by Perls and Perls/DAB staining methods. The seeds were directly germinated on +Pi medium and grown for four days before they were transferred to +Pi or -Pi medium with shoots grown under light (SL) and root grown under light (RL) or in darkness (RD). We then used the Perls and Perls/DAB staining to assess Fe accumulations in roots at 1, 2, and 3 DAT. Previously, by conducting a time course analysis of the Fe accumulation patterns in roots, Wang et al. (2019) found that the degree of the inhibition of PR growth by Pi deficiency is not linked to the level of Fe in the stem cell niche and elongation zone, but the maturation zone. Therefore, we next mainly focused on the Fe accumulation patterns in maturation zone (The upper row of each treatment in **Figure 2**). Under RL conditions, both the Fe^3+^ (**Figure 2A**) and total Fe (**Figure 2B**) were obviously accumulated in the maturation zone of the Pi-deficient roots at 2 and 3 DAT, which was consistent with the previous report (Wang et al., 2019). Under RD conditions, however, the accumulations of Fe^3+^ (**Figure 2A**) and total Fe (**Figure 2B**) in maturation zone were apparently abolished in Pi-deficient roots compared with those of the seedlings grown in RL conditions.

**Figure 3:**
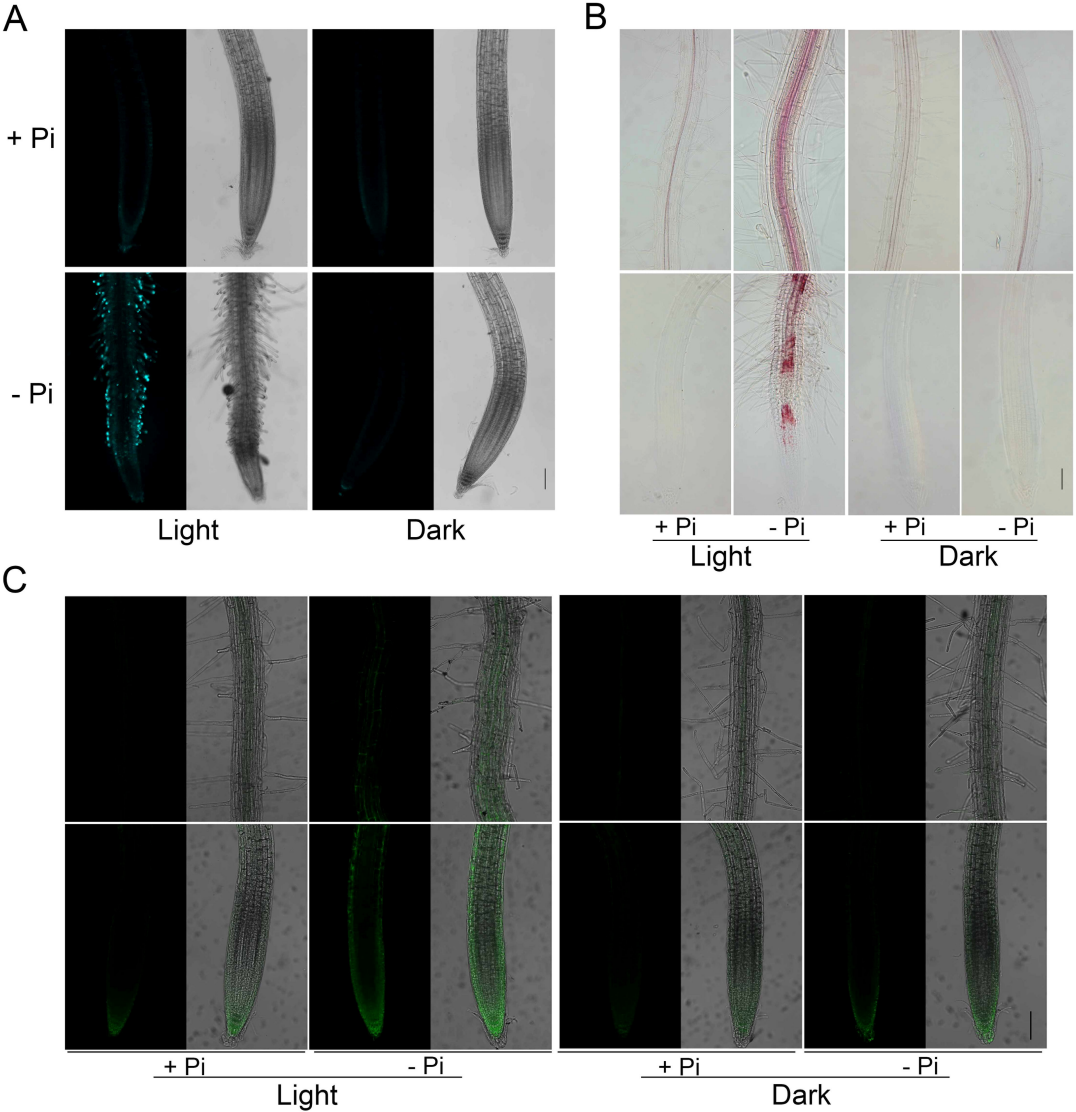
Callose, lignin, and ·OH staining in the roots on +Pi and –Pi media under light or in darkness. The seeds were directly germinated on +Pi medium and grown for four days before they were transferred to +Pi and -Pi medium under light or in darkness. Callose **(A)** and lignin **(B)** in roots were stained at 4 DAT. **(C)**·OH in the roots was assessed by HPF staining at 2 DAT. The upper and lower rows in each treatment of **(B, C)** are photographs of a part of the maturation zone and the root tip, respectively. The scale bar = 100 μm.

It was previously reported that in light-grown seedlings, LPR1-dependent accumulations of callose, lignin, and ·OH in root tips contribute to the inhibition of PR growth under -Pi condition (Williamson et al., 2001; Sanchez-Calderon et al., 2005; Müller et al., 2015; Ziegler et al., 2016; Zheng et al., 2019; Khandal et al., 2020). To further evaluate the impact of light exposure on the inhibition of PR growth under -Pi condition, we examined the accumulations of callose, lignin, and ·OH in the roots on +Pi and –Pi media under light or in darkness. The seeds were directly germinated on +Pi medium and grown for four days before they were transferred to +Pi or -Pi medium under SL+RL or SL+RD conditions. Callose and lignin staining assays were conducted at 4 DAT. Under RL conditions, the callose (**Figure 3A**) and lignin (**Figure 3B**) obviously deposited in Pi-deficient roots compared with those in Pi-sufficient roots, whereas the callose and lignin staining signals almost disappeared in the dark-grown roots. For ·OH detection, the seeds were directly germinated under +Pi condition for four days and then transferred to +Pi or -Pi medium with roots exposed to light or in the dark for another two days. The ·OH accumulation in roots were detected by HPF staining. We found that the light illumination-induced ·OH accumulation in roots under -Pi condition was reduced in darkness (**Figure 3C**). The above results together indicated that the accumulations of Fe, callose, lignin, and ·OH largely rely on light exposure on roots under Pi deficiency, which further confirmed the importance of root illumination in Pi deficiency-induced inhibition of PR growth.

### Blue light signaling pathway plays a minor role in regulating the inhibition of PR growth under -Pi condition

Blue light has been implicated in regulating Pi starvation-induced inhibition of PR growth (Zheng et al., 2019; Yeh et al., 2020), and long-distance blue light signaling components, CRY1/CRY2 and HY5, contribute to regulating this process by directly activating the expression of *LPR1* (Gao Y. Q. et al., 2021). We next investigated the function of CRY1/CRY2 and HY5 in the Pi deficiency-induced inhibition of PR growth. The seeds of the WT, *cry1cry2*, *hy5*, and *lpr1lpr2* were directly germinated on +Pi and -Pi media and PR lengths were evaluated at 8 DAG. On +Pi medium, the PR lengths of *cry1cry2* and *hy5* were significantly decreased compared to that of the WT and *lpr1lpr2* (**Supplementary Figures S1A, B**). The morphologies of the root tips of *cry1cry2*, *hy5*, and *lpr1lpr2* were comparable to that of the WT (**Supplementary Figure S1C**). Under -Pi condition, the PR lengths of *cry1cry2* and *hy5* were slightly longer than that of the WT, while *lpr1lpr2* showed a much longer PR length that was similar to itself grown on +Pi medium (**Supplementary Figures S1A, B**). The relative root lengths (average PR length of –Pi *vs*. +Pi) of *cry1cry2* (51%) and *hy5* (42%) were higher than that of the WT (17%), but lower than that of the *lpr1lpr2* (95%) (**Supplementary Figure S1B**), indicating that the *cry1cry2* and *hy5* were partially insensitive to Pi deficiency in terms of PR length. However, the morphologies of the root tips of *cry1cry2* and *hy5* were comparable to that of the WT, whereas the *lpr1lpr2* showed the similar root tip morphology with itself grown under +Pi condition (**Supplementary Figures S1C, D**).

**Figure 4:**
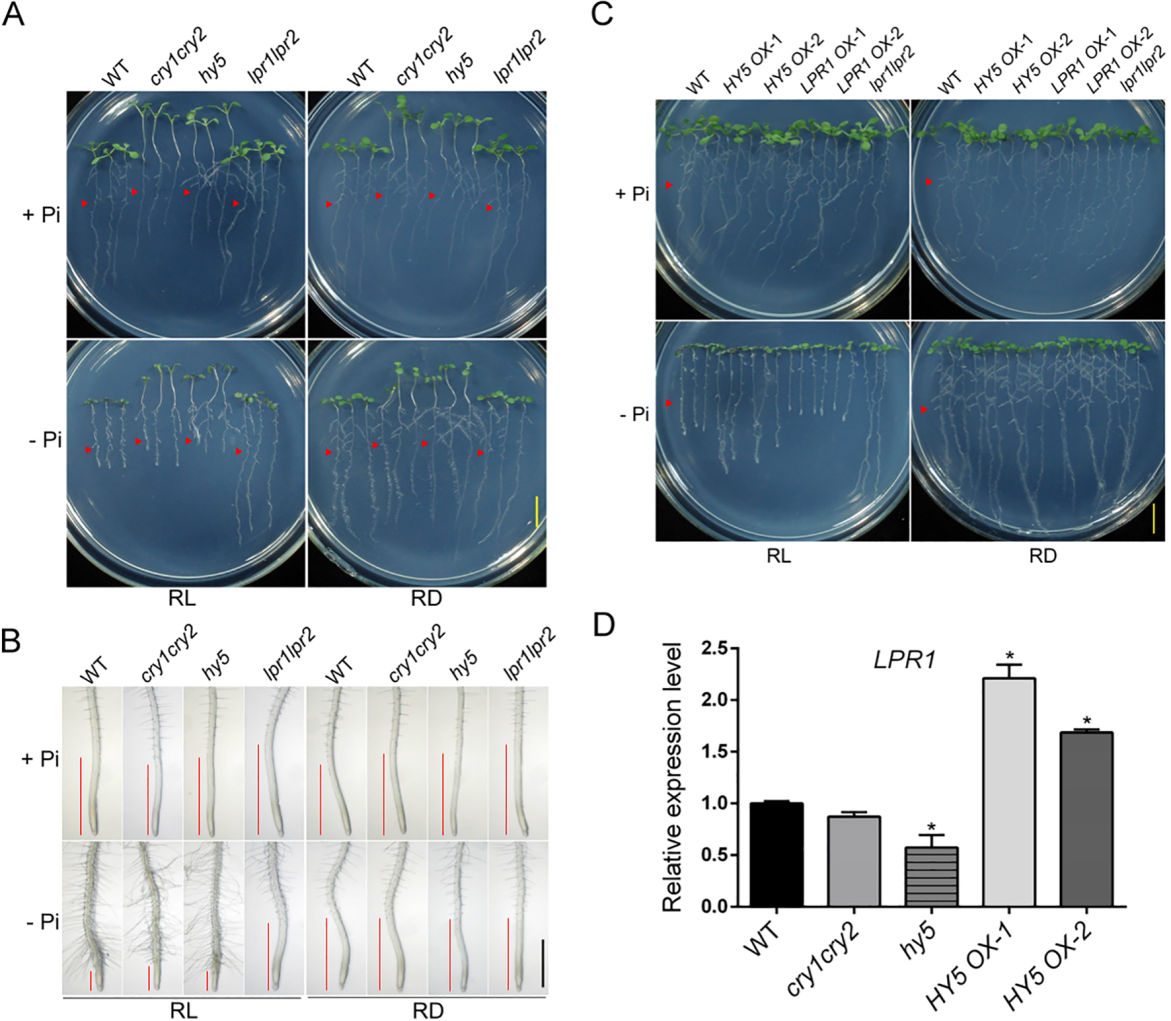
The role of light signaling pathway in Pi deficiency-induced inhibition of PR growth. **(A)** The 4-day-old +Pi seedlings of the WT, *cry1cry2*, *hy5*, and *lpr1lpr2* were transferred to +Pi or –Pi medium and grown for another four days with the shoots grown under light and roots grown under light or in darkness. RL: root under light; RD: root in dark. The red triangles indicate the position of the root tips before transfer. The scale bar = 1 cm. **(B)** The morphologies of the root tips of the seedlings shown in **(A)**. The red lines indicate the meristematic and elongation zones. The scale bar = 1 mm. **(C)** The 4-day-old +Pi seedlings of the WT, *HY5 OX-1*, *HY5 OX-2*, *LPR1 OX-1*, *LPR1 OX-2*, and *lpr1lpr2* were transferred to +Pi or –Pi medium and grown for another four days with the shoots grown under light and roots grown under light or in darkness. The red triangles indicate the root length before transfer. The scale bar = 1 cm. **(D)** RT-qPCR analyses of the expression of the *LPR1* gene in the seedlings in **(C)** under RL condition. *ACTIN2* was used as an internal control. The experiments were repeated three times, and representative results are shown. The values are means ± SD of three samples each with three technical replicates. Asterisks indicate significant differences from the WT (Student’s *t* test, *p < 0.01).

To avoid the influence of extremely low Pi environment on the root development of seedlings, we further performed the transferring experiment. The seeds of the WT, *cry1cry2*, *hy5*, and *lpr1lpr2* were directly germinated on +Pi medium and grown for four days before they were transferred to +Pi or -Pi medium and grown for another four days under SL+RL or SL+RD conditions. Under RL conditions, the PR length of *cry1cry2* and *hy5* were reduced to around half of the WT under +Pi condition. On –Pi medium, *cry1cry2* displayed similar PR lengths with WT, while *hy5* showed slightly reduced PR length compared to the WT (**Figure 4A**; **Supplementary Figure S2A**). The relative root lengths of *cry1cry2* (76%) and *hy5* (54%) were higher than that of the WT (46%), but lower than that of the *lpr1lpr2* (100%) (**Supplementary Figure S2A**), indicating that consistent with the direct germination assay (**Supplementary Figure S1**), the PR length of *cry1cry2* and *hy5* were partially insensitive to Pi deficiency. The Pi deficiency-induced morphological changes of the root tips of *cry1cry2* and *hy5* were more comparable to that of the WT but not *lpr1lpr2* (**Figure 4B**; **Supplementary Figure S2C**). Under RD conditions, the Pi deficiency-induced inhibition of PR growth and the morphological changes of the root tips of the WT, *cry1cry2*, and *hy5* were completely abolished (**Figures 4A, B**; **Supplementary Figures S2B, D**).

We next determined the Pi deficiency-induced accumulations of Fe, callose, lignin, and ·OH in the WT, *cry1cry2*, *hy5*, and *lpr1pr2*. The seeds were germinated on +Pi medium and grown for four days before they were transferred to +Pi and –Pi media with SL+RL or SL+RD. At 4 DAT, under RL condition, a large quantity of Fe^2+^ (**Supplementary Figure S3**) and total Fe (**Supplementary Figure S4**) accumulated in the maturation zone of the roots of the WT, *cry1cry2*, and *hy5*, but not *lpr1lpr2*, on -Pi medium. These accumulations almost disappeared when seedlings were grown in the dark. The same results could also be applied to the accumulations of callose, lignin, and ·OH (**Supplementary Figures S5-S7**). Overall, the Pi deficiency-induced accumulations of Fe, callose, lignin, and ·OH in *cry1cry2* and *hy5* were similar with that in the WT, but not that in *lpr1lpr2*.

Using protoplast assays, Gao Y. Q. et al. (2021) indicated that the overexpression of a single *HY5* gene was sufficient to upregulate *LPR1* expression. Based on this result, one would predict that the *HY5*-overexpressing (*HY5 OX*) line would have enhanced expression of *LPR1* and have a hypersensitive PR phenotype similar to that of the *LPR1-*overexpressing line (*LPR1 OX)* lines on –Pi medium as previously reported (Müller et al., 2015; Wang et al., 2019; Zheng et al., 2019). We then examined the phenotypes of two *HY5 OX* lines that had short hypocotyls, indicating that the overexpressed HY5 proteins were functional (**Supplementary Figure S8**). The 4-day-old Pi-sufficient seedlings of the WT, *HY5 OX-1*, *HY5 OX-2*, *LPR1 OX-1*, *LPR1 OX-2*, and *lpr1lpr2* were transferred to +Pi or –Pi medium and grown for another four days under SL+RL or SL+RD conditions. Under RL condition, we found that the *HY5-OX* lines had only a slightly enhanced expression of *LPR1* (a two-fold increase) (**Figure 4D**) and did not display the hypersensitive PR phenotype to Pi deficiency as *LPR1 OX* lines (**Figure 4C**; **Supplementary Figure S9A**). Interestingly, the *HY5 OX* lines were even more insensitive than the WT to Pi deficiency. Like that of the WT, the Pi deficiency-induced inhibition of PR growth of *HY5 OX* lines and *LPR1 OX* lines was largely suppressed in the dark (**Figure 4C**, **Supplementary Figure S9B**).

Taken together, these results indicated that although the functional disruption of blue light signaling components, CRY1/CRY2 and HY5, result in partial insensitivity of the inhibition of PR growth in response to Pi deficiency, this phenotype cannot be properly explained by their regulation of the expression of *LPR1* and by LPR1-dependent accumulations of callose, lignin, and ·OH in root tips under our experimental conditions.

### Genome-wide identification of light- and dark-affected phosphate starvation responsive genes

**Figure 5:**
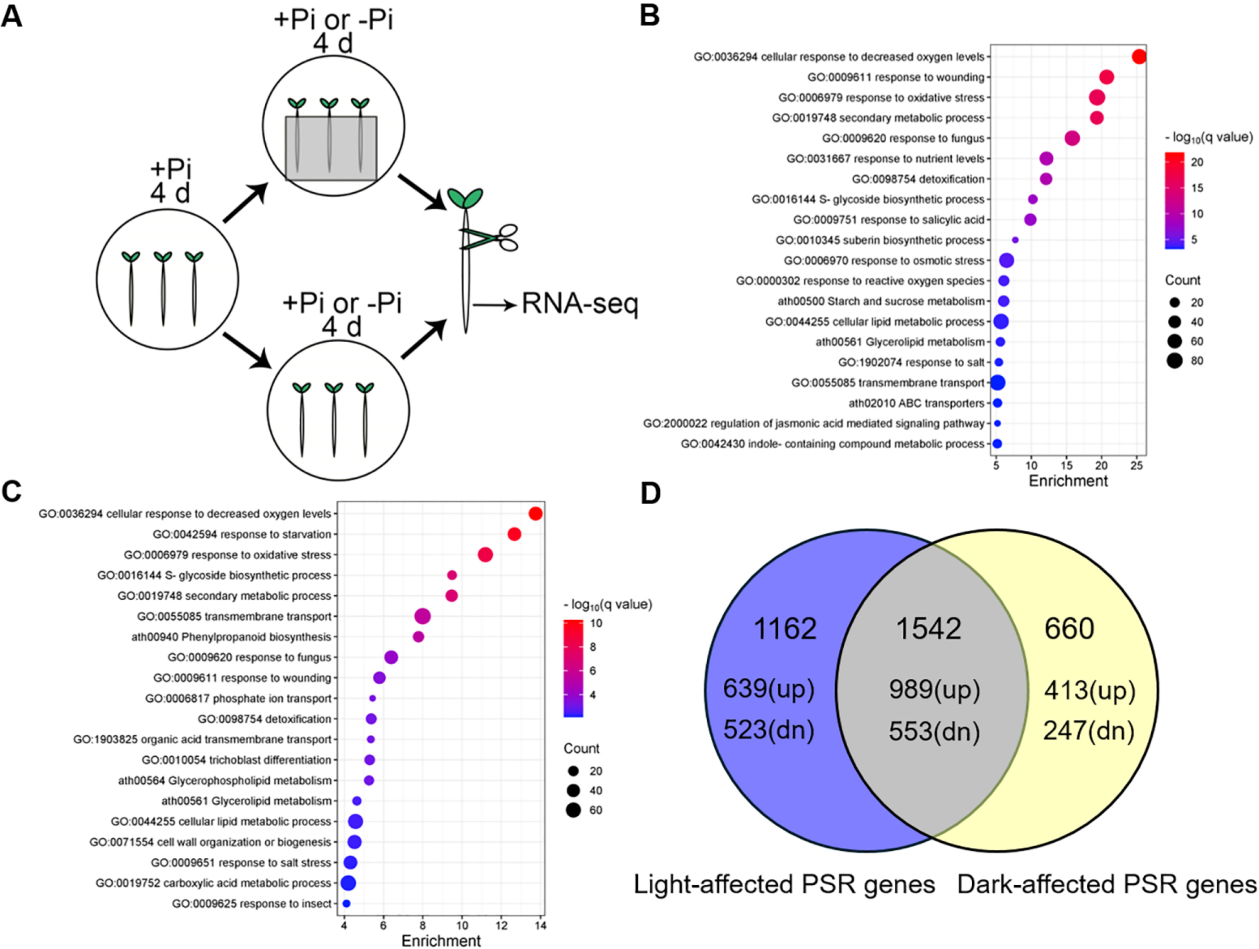
Genome-wide identification of light- and dark-affected phosphate starvation responsive (PSR) genes. **(A)** The schematic diagram depicts the experimental procedure in this study. The 4-day-old +Pi seedlings were transferred to +Pi or –Pi medium with their shoots grown under light and roots grown under light or in darkness for another four days. Then the roots were collected for RNA extraction and RNA-seq analyses. **(B)** The GO analysis of the light-affected PSI genes. **(C)** The GO analysis of the dark-affected PSI genes. **(D)** The common and specific light- and dark-affected PSR genes. Blue circle represents the light-affected PSR genes, while yellow circle represents the dark-affected PSR genes. “up” in the brackets represents number of the light or dark-affected PSI genes, while “dn” in the brackets represents the number of the light or dark-affected PSS genes.

Light affects phosphate starvation responses not only reflected in regulating Pi deficiency-induced inhibition of PR growth under blue light, but also in regulating the transcriptomic changes, for example, red/far-red light regulates the expression of Pi starvation-induced (PSI) genes by activation of PHR1 (Liu et al., 2017), whereas simulated shade conditions compromised Pi starvation responses via repress PHR1 activity by JAZ proteins (Sun et al., 2023). On the other hand, photosynthate sucrose is a global regulator of plant responses to Pi starvation and 73% of the genes that are induced by Pi starvation in WT plants can be induced by elevated levels of sucrose in *hps1* mutants (Lei et al., 2011). To further elucidate the impact of light on the transcriptional changes under -Pi, we conducted transcriptomic analyses of the roots grown on +Pi and -Pi media under light or in darkness. The seeds were directly germinated under +Pi condition for four days and transferred to +Pi or -Pi medium with roots exposed to light or in darkness for another four days and then the roots were collected for total RNA extraction (**Figure 5A**). These batches of RNAs were then subjected to sequencing using next generation sequencing technology. Three biological replicates were used for all genotypes.

We first analyzed the effects of light on the expression of genes in roots grown on +Pi medium. Using |Log_2_FC| ≥ 1 (differential expression level greater than 2 folds) and FDR (false discovery rate) ≤ 0.01 as cutoff, we identified 86 differentially expressed genes (DEG) that were upregulated and 186 DEGs that were downregulated in the roots grown in the dark compared with that grown under light (**Supplementary Figure S10A**; **Supplementary Table S2A**). Gene Ontology (GO) analysis showed that most of these DEGs were involved in photosynthesis and response to light stimulus which is consistent with former results (**Supplementary Figure S10B**) (Silva-Navas et al., 2015; Qu et al., 2017; Zhang et al., 2019).

We then determined the effects of light on the expression of PSR genes. When roots were exposed to light, we identified 2704 PSR genes by comparison of the gene expression levels between roots grown under –Pi-light and +Pi-light, including 1628 PSI genes and 1076 Pi starvation-suppressed (PSS) genes (**Supplementary Figure S11A**, **Supplementary Table S2B**). These PSR genes were hereafter referred to as light-affected PSR genes. GO analyses showed that most light-affected PSI genes were enriched in cellular response to decreased oxygen levels, response to wounding, response to oxidative stress, and response to nutrient levels (**Figure 5B**), while most light-affected PSS genes were enriched in photosynthesis, response to light intensity, cellular response to sucrose starvation, intracellular iron ion homeostasis (**Supplementary Figure S11B**). When roots were grown in the dark, we identified 2202 PSR genes by comparison of the gene expression levels between roots grown under –Pi-dark and +Pi-dark, which included 1399 PSI genes and 803 PSS genes (**Supplementary Figure S12A**, **Supplementary Table S2C**). These PSR genes were defined as dark-affected PSR genes. GO analyses indicated that most dark-affected PSI genes were enriched in cellular response to decreased oxygen levels, response to starvation, phosphate ion transport, cellular lipid metabolic process, trichoblast differentiation (**Figure 5C**), while most dark-affected PSS genes were enriched in photosynthesis, response to absence of light, nitrogen metabolism, intracellular iron ion homeostasis (**Supplementary Figure S12B**).

### The common and specific light- and dark-affected PSR genes

To identify the common and specific light- and dark-affected PSR genes, a Venn diagram was drawn by comparison of the expression levels between light- and dark-affected PSR genes. We obtained 1542 common PSR genes between light- and dark-affected PSR genes, 1162 PSR genes that were specifically affected by light conditions and 660 PSR genes that were specifically affected by dark conditions (**Figure 5D**).

For the 1542 common PSR genes that were both affected by light and dark conditions, there were 989 PSI genes and 553 PSS genes (**Figure 5D**). The GO analysis indicated that most common PSI genes were enriched in cellular response to decreased oxygen levels, response to nutrient levels, S-glycoside biosynthetic process, phosphate ion transport (**Supplementary Figure S13A**; **Supplementary Tables S2B, C**), and most common PSS genes were enriched in photosynthesis, response to light intensity, photosystem II assembly (**Supplementary Figure S13B**; **Supplementary Tables S2B, C**). Next, we analyzed the PSR genes that were specifically affected by light and dark conditions. We identified 639 specific light-affected PSI genes and 523 specific light-affected PSS genes (**Figure 5D**). The GO analysis of 639 specific light-affected PSI genes showed that most of these genes were enriched in response to wounding, response to fungus, defense response to other organism, response to salicylic acid, indole-containing compound metabolic process (**Supplementary Figure S13C**; **Supplementary Table S2B**). Most specific light-affected PSS genes were enriched in photosynthesis and water transport (**Supplementary Figure S13D**; **Supplementary Table S2B**). Finally, we identified 413 specific dark-affected PSI genes and 247 specific dark-affected PSS genes (**Figure 5D**). GO analysis suggested that most specific dark-affected PSI genes were enriched in root hair elongation, wax biosynthetic process, flavonoid biosynthesis (**Supplementary Figure S13E**; **Supplementary Table S2C**), and most specific dark-affected PSS genes were enriched in nitrogen metabolism, cellular response to hypoxia and so on (**Supplementary Figure S13F**; **Supplementary Table S2C**).

### Expression profile of the light- and dark-affected PSR genes

To give a global view of how light and dark conditions affect the transcriptomic changes under +Pi and –Pi media, we drew a heatmap using the expression levels of all PSR genes (obtained by comparisons of roots grown under -Pi-light *vs*. +Pi-light, -Pi-dark *vs*. +Pi-dark, -Pi-dark *vs*. +Pi-light) among four types of treatments (**Supplementary Tables S2B-D**).

**Figure 6:**
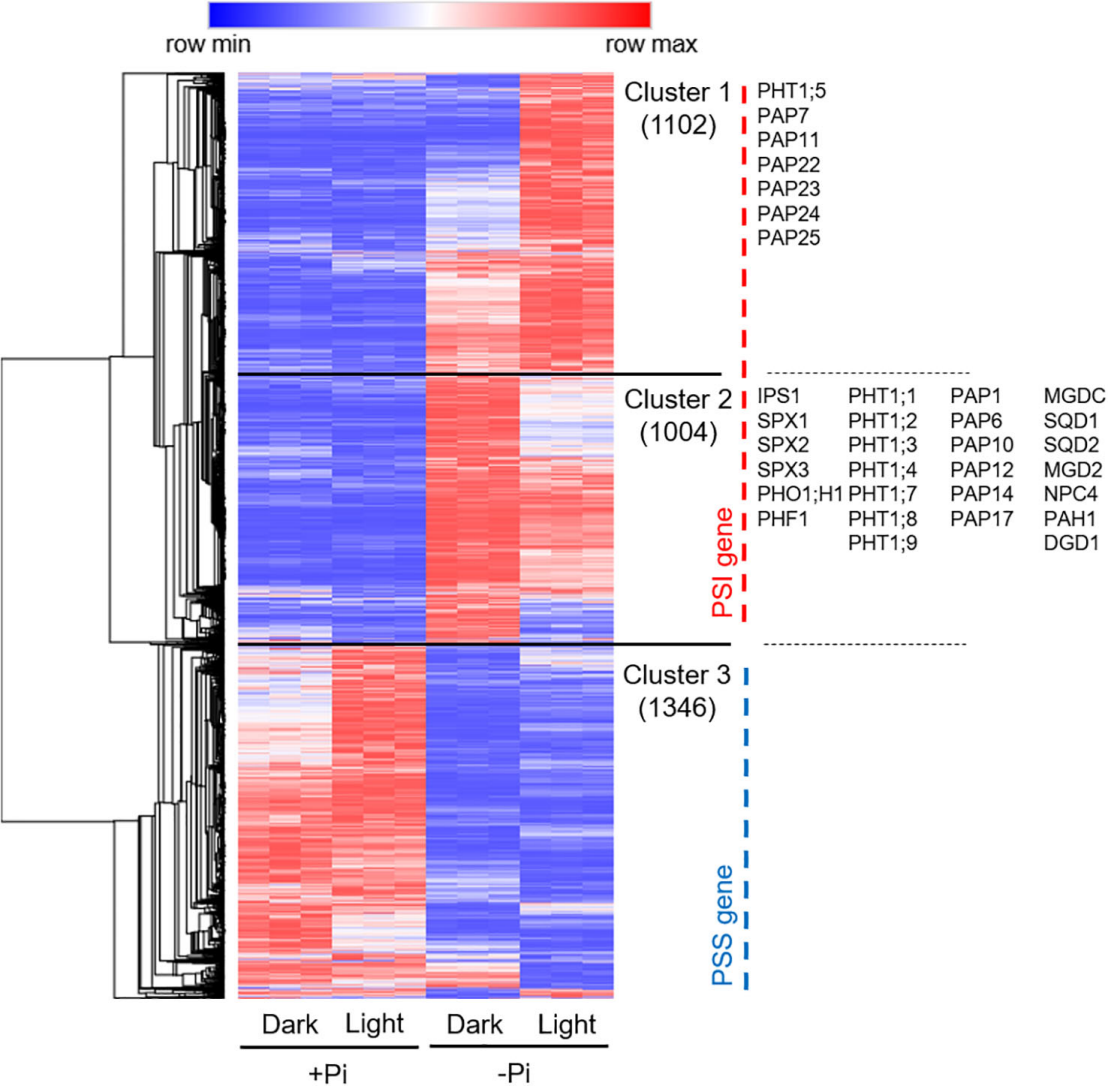
Expression profile of the light- and dark-affected PSR genes. The expression levels of all PSR genes were used to draw the heatmap. Three clusters were obtained by hierarchical clustering, among which genes in cluster 1 and 2 belong to PSI genes while genes in cluster 3 belong to PSS genes. The TPM (transcripts per million) values used in the heatmaps have been standardized by min-max standardization. The gene number of each cluster were shown in brackets. The positions of the known or putative PSI genes were indicated at the right side of the heatmap.

Hierarchical clustering was conducted and presented in the heatmap (**Figure 6**; **Supplementary Table S3**). By hierarchical clustering analysis, we obtained three clusters, among which cluster 1 and 2 represented PSI genes while cluster 3 represented PSS genes. We found that under -Pi condition, light had more impact on the expression of PSI genes than PSS genes. For 1102 PSI genes in cluster 1, the expression of most genes was upregulated by Pi starvation in the dark and was further increased when roots were exposed to light illumination. GO analysis suggests that these genes were highly enriched in response to wounding and fungus, cellular response to decreased oxygen levels, glucosinolate biosynthesis from methionine, response to salt, and hormone-related processes, such as salicylic acid, auxin, abscisic acid, and jasmonic acid (**Figure 6**; **Supplementary Figure S14A**). For 1004 PSI genes in cluster 2, the expression of the genes was upregulated in the dark on -Pi medium, whereas the upregulation was partially decreased when roots were exposed to light. GO analysis suggested that these genes were highly enriched in cellular response to phosphate starvation, glycerophospholipid metabolism, trichoblast differentiation, inositol phosphate metabolism, sulfolipid metabolic process, cell wall organization or biogenesis (**Figure 6**; **Supplementary Figure S14B**). For 1346 PSS genes in cluster 3, light has more effect on the expression of these genes on +Pi medium than –Pi medium. GO analysis suggested that these genes were enriched in photosynthesis, iron ion transport, cellular response to sucrose starvation and so on (**Figure 6**; **Supplementary Figure S14C**).

To elucidate the impact of light exposure on the expression of typical PSI genes (Bustos et al., 2010; Wang et al., 2024), we selected a group of PSI genes that are known or putative to be involved in various Pi starvation responses, such as Pi signaling (*IPS1*, *SPX1*, *SPX2*, *SPX3*, *PHF1*, *PHO1;H1*), Pi transport (*PHT1;1*, *PHT1;2*, *PHT1;3*, *PHT1;4*, *PHT1;5*, *PHT1;7*, *PHT1;8*, *PHT1;9*), Pi remobilization (*PAP1*, *PAP6*, *PAP7*, *PAP10*, *PAP11*, *PAP12*, *PAP14*, *PAP17*, *PAP22*, *PAP23*, *PAP24*, *PAP25*), and phospholipid remodeling (*MGDC*, *SQD1*, *SQD2*, *MGD2*, *NPC4*, *PAH1*, *DGD1*). We found that most of these genes belong to cluster 2, while a few of them belong to cluster 1 (**Figure 6**; **Supplementary Table S3**).

Taken together, these results indicated that light has a substantially promotion on Pi deficiency-induced cellular response to stress (biotic and abiotic) and phytohormone-related processes (SA, auxin, ABA, JA), while has a suppression of genes involved in cellular response to Pi starvation, such as Pi signaling, Pi transport, Pi remobilization, and phospholipid remodeling.

### Light has both positive and negative effects on the expression of known or putative PSI genes under -Pi condition

**Figure 7:**
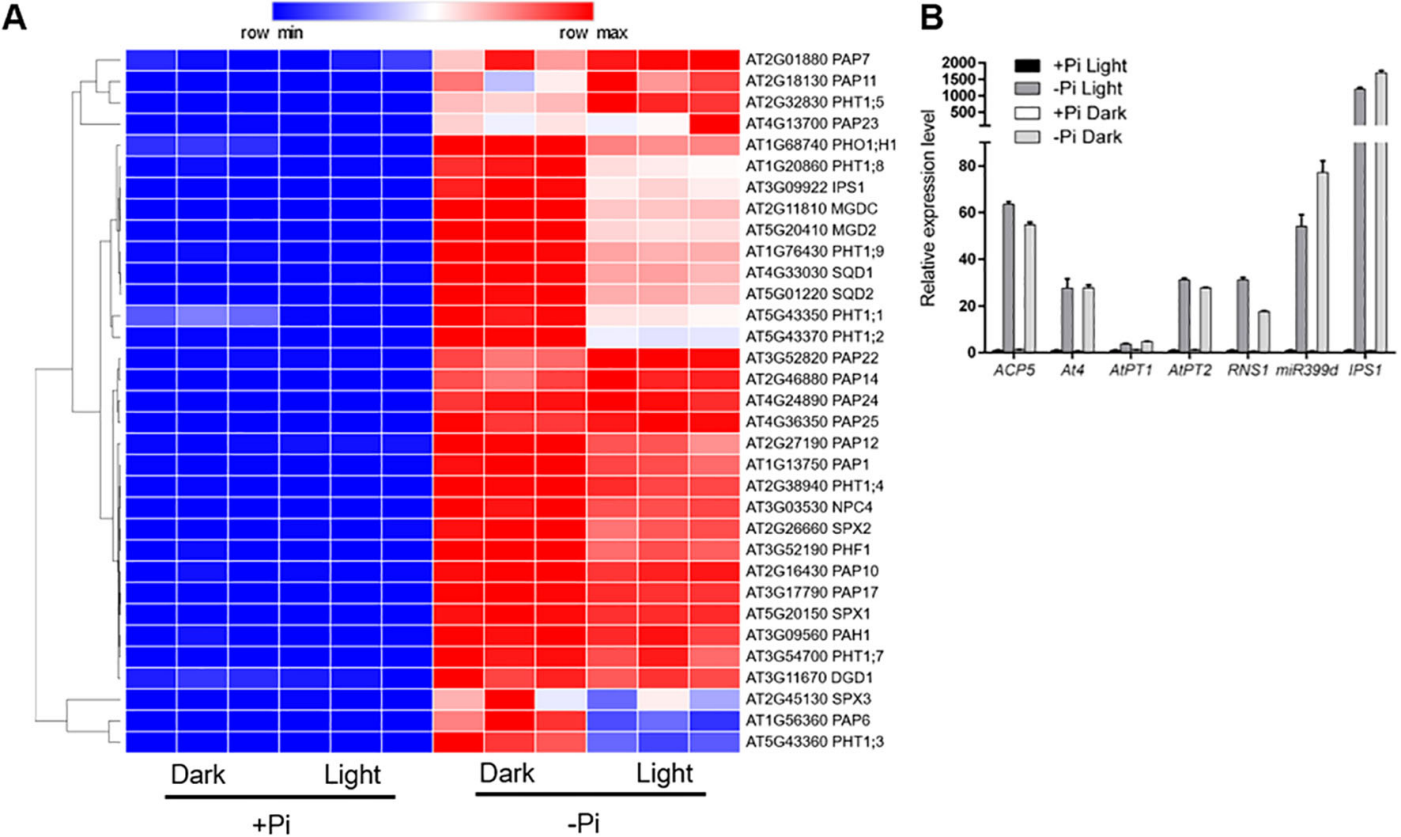
Light has both positive and negative effects on the expression of known or putative PSI genes under -Pi condition. **(A)** The heatmap of 33 known or putative PSI genes under light or in darkness. A relative color scheme uses the minimum and maximum values in each row to convert values to colors. **(B)** RT-qPCR analyses of the expression level of seven hallmark PSI genes under different conditions in roots. *ACTIN2* was used as an internal control. The experiments were repeated three times, and representative results are shown. The values are means ± SD of three samples each with three technical replicates.

To further reveal the light effects on the expression of above-mentioned typical PSI genes, we drew a heatmap of these genes by their expression levels (**Figure 7A**). In this heatmap, we found that the effects of light on the expression of the typical PSI genes can be summarized into three categories: (1) light exposure promotes the expression of the PSI genes, such as *PHT1;5*, *PAP7/11/23*; (2) light exposure inhibits the expression of the PSI genes, such as *IPS1*, *PHT1;1*, *SPX3*; (3) light exposure has no obvious effect on the expression of the PSI genes, such as *SPX1*, *SPX2*, and *PHF1* (**Figure 7A**). To confirm this, we conducted RT-qPCR analysis of seven hallmark PSI genes, including two non-coding transcripts, *IPS1* and *AT4* (Burleigh and Harrison, 1999); a microRNA, *miR399d* (Fujii et al., 2005); two high-affinity phosphate transporters, *AtPT1* (*Pht1;1*) and *AtPT2* (*Phtl;4*) (Muchhal et al., 1996); a ribonuclease, *RNS1* (Bariola et al., 1994); and an acid phosphatase, *ACP5* (*AtPAP17*) (del Pozo et al., 1999). We found that light has positive effects on the expression of *ACP5*, *AtPT2*, and *RNS1*, whereas has negative effects on the expression of *miR399d* and *IPS1* (**Figure 7B**). These results demonstrated that light has both positive and negative effects on the expression of known or putative PSI genes under -Pi condition.

### Light enhances local and systemic phosphate starvation responses

**Figure 8:**
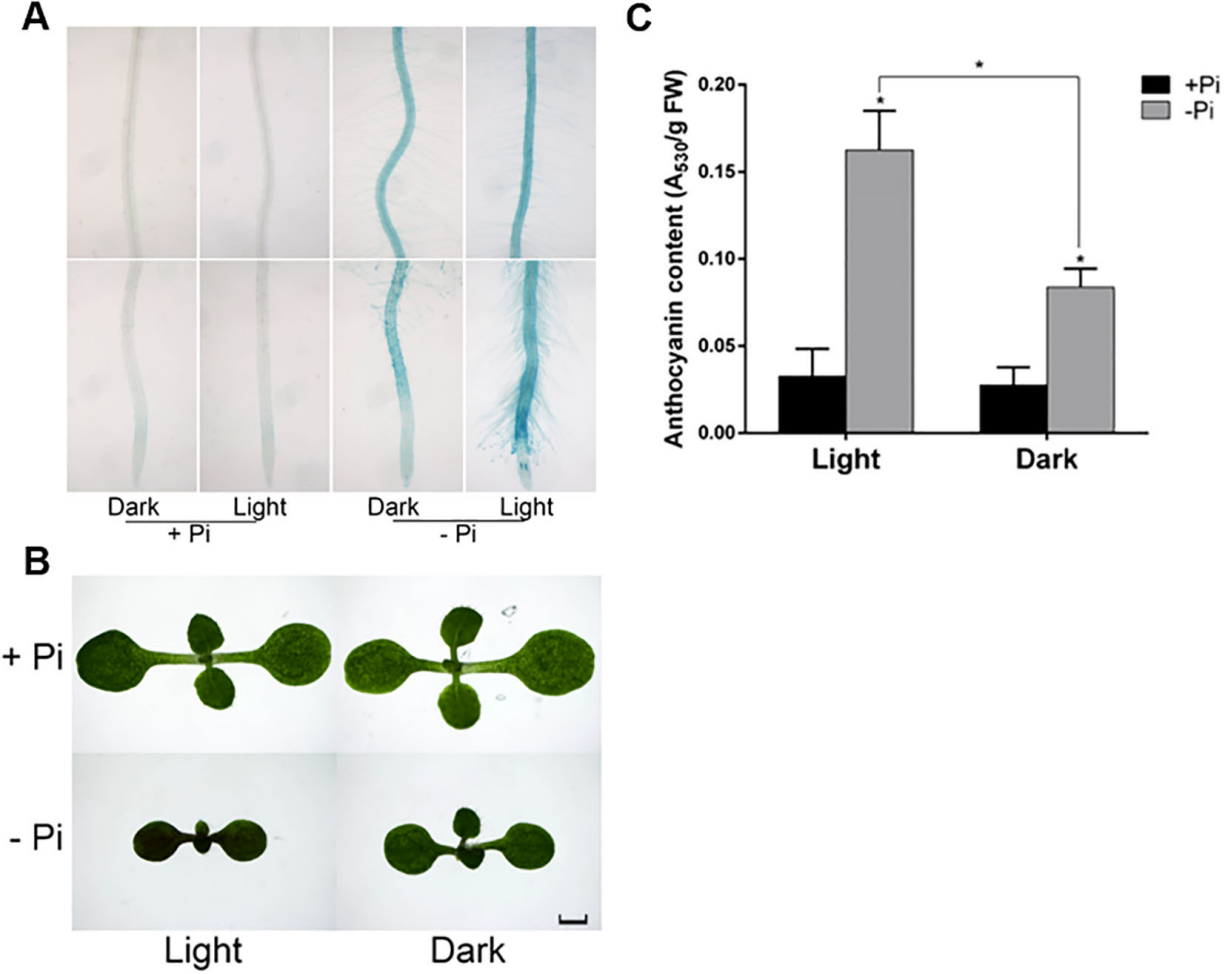
The root-associated APase activity and anthocyanin accumulation of the WT on +Pi and –Pi media under light or in darkness. The WT seedlings grown on +Pi for four days and then transferred into +Pi or -Pi medium for another four days with shoots grown under light and roots grown under light or in the dark. **(A)** Root-associated APase activity were detected by BCIP staining. Anthocyanin accumulation were detected by microscopy **(B)** and A_530_ absorption **(C)**. In **(C)**, The experiments were repeated three times, and representative results are shown. The values are means ± SD of three samples each with three technical replicates. Asterisks indicate significant differences from the WT (Student’s *t*-test, *p <0.05). The scale bar = 1 mm.

Given light has a profound influence on the transcriptomic changes under –Pi condition, we wondered whether it affects other Pi starvation responses. Induction and secretion of APases on the root surface is one of the hallmark responses of plants to Pi starvation. Among multiple purple acid phosphatases in Arabidopsis, PAP10, is predominantly associated with the root surface after its secretion (Wang et al., 2011). Root surface-associated APase activity can be detected by histochemical staining using a substrate of APase, BCIP (5-bromo-4-chloro-3-indolyl phosphate). The product of the enzyme reaction forms a blue precipitate. We next evaluated the APase activity in the root surface by BCIP staining. We found that acid phosphatase activity on the root surface was highly induced on -Pi medium in the dark and this induction was apparently further increased when roots were exposed to light condition (**Figure 8A**).

As light enhanced the root surface-associated APase activity in roots, we next wondered whether light illumination on roots could stimulate systemic responses in shoots. Anthocyanin accumulation is acknowledged as a phenotypic indicator of Pi starvation. We then determined the anthocyanin accumulation in shoots when roots were grown on +Pi and -Pi media under light or in darkness. The seeds were grown on +Pi medium for four days and transferred into +Pi and –Pi media for another four days under SL+RL or SL+RD conditions before anthocyanin accumulation was detected. We found that a large quantity of anthocyanins accumulated in shoots on -Pi medium under light and the accumulation was largely decreased when roots were grown in darkness (**Figures 8B, C**).

Taken together, these results suggested that light exposure on roots results in increased Pi starvation responses, such as root surface-associated APase activity; it also triggers systemic stress responses in shoots of seedlings, such as anthocyanin accumulation, by an as yet undetermined mechanism.

## Discussion

The inhibition of PR growth is a major response of Arabidopsis to Pi deficiency. In the past decades, by identifying a group of mutants that are insensitive to Pi deficiency-induced inhibition of PR growth, there is a common view that Fe accumulation and malate in roots is critical to this process (Svistoonoff et al., 2007; Müller et al., 2015; Balzergue et al., 2017; Mora-Macías et al., 2017; Wang et al., 2019). Our laboratory further showed that direct blue light illumination on roots is both required and sufficient for the Pi deficiency-induced inhibition of PR growth (Zheng et al., 2019). Another study revealed that the inhibition of root elongation by -Pi requires blue light signal perception at the shoot and transduction to the root (Gao Y. Q. et al., 2021). The discrepancy between this work and our study raised questions that which part (shoots or roots) contributes to Pi deficiency-induced inhibition of PR growth, and that whether blue light signaling pathway is involved in this process.

After analyses of the experimental data provided in Gao Y. Q. et al. (2021), we considered that the experimental results did not disprove the notion that light illumination on roots is required to trigger the Pi deficiency-induced inhibition of PR growth. Meanwhile, light shielding of shoots did not abolish this responsiveness. In this work, to address whether shoots or roots are critical for Pi deficiency-induced inhibition of PR growth, we conducted detailed light shielding assays to separate the role of shoots and roots in the inhibition of PR growth under -Pi. We found that compared to the inhibition of PR growth in Pi-deficient SL+RL seedlings, the Pi deficiency-induced inhibition of PR growth were abolished in SL+RD seedlings, suggesting that the light illumination on roots is required for this process; on the other hand, compared to the SD+RD seedlings, the Pi deficiency-induced PR growth inhibition and typical morphological changes of root tips, especially the reduced meristem size, was observed for SD+RL seedlings under -Pi condition, indicating that the light illumination on roots is sufficient for this process (**Figure 1**). Notably, when shoots were placed in darkness (SD+RD and SD+RL), the PR lengths of these seedlings were decreased to about one half under +Pi condition in contrast to that of SL+RL seedlings under +Pi (**Figure 1B**). These could be attributed to two possible reasons: (1) Fewer photosynthates are delivered to roots to support their growth due to the lack of photosynthesis. By addition of sucrose into medium, this hypothesis can be easily tested in future; (2) Light illumination on shoots promotes PR elongation by long-distance shoot-root communication, which was repressed by shielding shoots from light. Previous studies have shown that COP1 and HY5 are involved in light-mediated PR growth by regulation of auxin transporters (Sassi et al., 2012) and the balance of C/N (Chen et al., 2016). Last but not least, the contrasting results obtained by Gao Y. Q. et al. (2021) and our laboratory (Zheng et al., 2019) might be resulted from different experimental designs (direct germination assay or transferring experiment), medium formulas (Hoagland medium or 1/2 MS medium), Pi contents (+Pi: 250μM, -Pi: 50μM or +Pi: 625μM, -Pi: 0μM), and environmental conditions (light intensity of 100 or 200 μmol m^-2^ s^-1^).

The initial evidence for the working model proposed by Gao Y. Q. et al. (2021) was that the blue light signaling mutants *cry1cry2* and *hy5* had a reduced expression of *LPR1* (the mutants still retained 20-30% of expression) and were completely insensitive to Pi deficiency-induced inhibition of PR growth. In our work, we found that the PR growth of cry*1cry2* and *hy5* was still partially inhibited by Pi deficiency and that the degree of inhibition depended on the growth conditions (seeds were germinated on +Pi medium and grown for four days before they were transferred to –Pi medium for another four days, or seeds were germinated on –Pi medium and grown for eight days). Consistently, the partial inhibition of PR growth of *cry1cry2* and *hy5* on -Pi medium has also been reported by Yeh et al. (2020). We also noticed that the morphological changes of the root tips of *cry1cry2* and *hy*5 did not differ from those of the WT under Pi deficiency (**Figure 4B**, **Supplementary Figure S2C**). Furthermore, like WT root tips, the root tips of *cry1cry2* and *hy5* had enhanced Fe accumulation, callose deposition, lignin formation, and ROS accumulation under Pi deficiency (**Supplementary Figures S3-7**). These responses were largely suppressed when roots were grown in the dark (**Figure 4B**, **Supplementary Figures S2D**, **S3-S7**).

Although Gao Y. Q. et al. (2021) showed that *hy5* had reduced expression of *LPR1* in roots, there was no direct evidence supporting the inference that the insensitivity of *hy5* to Pi deficiency was caused by the reduced expression of *LPR1*. In our work, we found that the *HY5 OX* lines had about two-fold increased expression of *LPR1* (**Figure 4D**) and did not display the hypersensitive PR phenotype to Pi deficiency as *LPR1 OX* lines (**Figure 4C**; **Supplementary Figure S9A**). Interestingly, the *HY5 OX* lines were even more insensitive than the WT to Pi deficiency. The above results indicated that despite the PR growth of *cry1cry2* and *hy5* displayed partial insensitivity to Pi deficiency, these phenotypes cannot be explained by their regulation of the expression of *LPR1* and by LPR1-dependent Fe redox cycle in root tips under our experimental conditions. Instead, one could only conclude that the accumulation of HY5 in roots plays a role in maintaining the basal expression of *LPR1* under both Pi sufficiency and Pi deficiency (Gao Y. Q. et al., 2021).

Over the last 20 years, many Arabidopsis mutants with altered sensitivity of PR growth to Pi deficiency have been identified. Except for *lpr1*, *stop1*, *almt1*, *als3*, *star1*, *crr*, and *hyp1* (Svistoonoff et al., 2007; Balzergue et al., 2017; Dong et al., 2017; Mora-Macías et al., 2017; Clúa et al., 2024; Maniero et al., 2024), most of them, however, exhibited developmental abnormality under +Pi (Ma et al., 2003; Miura et al., 2005; Nacry et al., 2005; Jiang et al., 2007; Mayzlish-Gati et al., 2012; Singh et al., 2014). HY5 acts as a master regulator of light-mediated transcriptional regulatory hub that directly or indirectly controls the transcription of approximately one-third of genes at the whole genome level (Xiao et al., 2022). It was reported that HY5 may regulate the PR growth by directly controlling the expression of auxin and BR pathway related genes (Li et al., 2020), and by regulating the light-responsive transcription of *miRNA163* (Li et al., 2021). Besides, *hy5* has an insensitive PR growth phenotype in thermomorphogenesis process (Lee et al., 2021). Considering the short PR lengths of *cry1cry2* and *hy5* under normal conditions, we assumed that CRY1/CRY2 and HY5 possess pleotropic effects in regulating PR growth and development, and that the partial insensitivity of PR growth of *cry1cry2* and *hy5* in response to Pi deficiency was independent of their regulation of the expression of *LPR1*.

In fact, the photo-Fenton reaction not only occurs in Pi deficiency-induced inhibition of PR growth as a source of damage in transparent Petri dishes, it also takes place in the shoots of seedlings under normal condition. For example, the LPR1-mediated Fe^2+^ oxidation and blue light-triggered photo-Fenton reaction were observed in the translocation of Fe in the xylem for Fe distribution in plants. A recent study revealed that LPR1 and LPR2 are required to oxidize Fe^2+^ and maintain Fe^3+^-citrate stability and mobility during xylem translocation against photoreduction (Xu et al., 2022). Therefore, we speculated that when we place Pi-deficient roots in light exposure, it triggers an existing mechanism of Fe distribution of shoots. Unlike the xylem with cell wall lignification and protoplast lysis in shoots, the root apical meristem is more fragile in response to damages, thereby resulting in Pi deficiency-induced inhibition of PR growth under light.

Light is one of the most important environmental signals and regulates many biological processes in plants. Previous studies reported that both light signaling pathway and photosynthate sucrose have a significant impact on transcriptional changes under –Pi (Lei et al., 2011; Liu et al., 2017). To further illustrate the light effects on transcriptional changes under –Pi, we analyzed the transcriptomic changes in roots on +Pi and -Pi media under light or in darkness (**Figure 5A**). We identified 1628 light-affected PSI genes and 1076 light-affected PSS genes, 1399 dark-affected PSI genes and 803 dark-affected PSS genes (**Supplementary Tables S2B, C**). By comparison of common and specific light- and dark-affected PSR genes, we found that among 1542 common PSR genes, most common PSI genes were enriched in cellular response to decreased oxygen levels, response to nutrient levels, S-glycoside biosynthetic process, phosphate ion transport (**Supplementary Figure S13A**), most common PSS genes were enriched in photosynthesis, response to light intensity, photosystem II assembly (**Supplementary Figure S13B**), which were consistent with previous transcriptomic studies under Pi starvation (Wu et al., 2003; Misson et al., 2005; Morcuende et al., 2007; Bustos et al., 2010; Osorio et al., 2019; Hani et al., 2021; Wang et al., 2023). For specific light-affected PSI genes, most of them were enriched in response to wounding, response to fungus, defense response to other organism, response to salicylic acid, indole-containing compound metabolic process (**Supplementary Figure S13C**); most specific light-affected PSS genes were enriched in photosynthesis and water transport (**Supplementary Figure S13D**). For specific dark-affected PSI genes, most of them were enriched in root hair elongation, wax biosynthetic process, flavonoid biosynthesis (**Supplementary Figure S13E**); most specific dark-affected PSS genes were enriched in nitrogen metabolism, cellular response to hypoxia (**Supplementary Figure S13F**). These results indicated that the transcriptomic changes of an array of PSR genes (specific light- and dark-affected PSR genes, including genes involved in stress and phytohormone-related processes, root hair elongation, nitrogen metabolism) are due to the light illumination on roots in transparent Petri dishes instead of Pi deficiency per se.

By drawing a heatmap and conducting hierarchical clustering analysis, we found that light exerted more effects on the expression of PSI genes than PSS genes. For PSI genes, light has both function of induction (cluster 1) and suppression (cluster 2). Those light-promoted genes in cluster 1 were highly enriched in response to wounding and fungus, cellular response to decreased oxygen levels, glucosinolate biosynthesis from methionine, response to salt, and hormone-related processes, such as salicylic acid, auxin, abscisic acid, and jasmonic acid (**Figure 6**; **Supplementary Figure S14A**). This result was similar with the GO enrichment of specific light-affected PSI genes. Consistently, Arabidopsis roots grown under light have been demonstrated to have altered responses to stresses (osmotic and salt) and phytohormones compared to those grown in the dark (Yokawa et al., 2013, 2014; Silva-Navas et al., 2015; Cabrera et al., 2021). The light-repressed genes in cluster 2 were highly enriched in cellular response to phosphate starvation, glycerophospholipid metabolism, trichoblast differentiation, inositol phosphate metabolism, sulfolipid metabolic process, cell wall organization or biogenesis (**Figure 6**; **Supplementary Figure S14B**). We also detected many known or putative PSI genes in cluster 2. By drawing a detailed heatmap for these typical PSI genes, we found that light has both induction and repression effects on the expression of these genes (**Figure 7A**). Earlier researches have shown that active light signaling is required for the induction of PHR1 expression and simulated shade conditions compromised Pi starvation responses via repress PHR1 activity by JAZ proteins (Liu et al., 2017; Sun et al., 2023). By transcriptomic analyses, we confirmed the upregulation of a series of PSI genes under light and also found the downregulation of PSI genes by light exposure, suggesting that there might have other unknown ways of repressing the expression of PSI genes on –Pi medium under light. Further study is still needed to clarify whether PHR1, or other components, are involved in this process.

By assessing the root surface-associated APase activity on roots and anthocyanin accumulation in shoots on +Pi and –Pi media under light or in darkness (**Figure 8**), we found that light affects Pi starvation responses both in local and systemic manners. The anthocyanin biosynthetic pathway is derived from the flavonoid pathway. Previous studies have shown that the flavonoid pathway is highly induced in illuminated shoots and roots (Silva-Navas et al., 2016; Qu et al., 2017; Miotto et al., 2019) and it has been reported that flavonoids are systemically mobile (Buer et al., 2007). Therefore, it is plausible that root-synthesized anthocyanin precursors are transported to the shoot, increasing anthocyanin content, which will be interesting to determine the underlying mechanism.

## Conclusion

This work clarified that light illumination on roots, but not shoots, is both required and sufficient for Pi deficiency-induced inhibition of PR growth. Besides, the Pi deficiency-induced accumulations of Fe, callose, lignin, and ·OH are attenuated in roots grown in darkness. Blue light signaling pathway plays a minor role in regulating the inhibition of PR growth under -Pi condition. Light promotes the expression of a large number of genes involved in stress and phytohormone-related processes and has both upregulated and downregulated effects on the expression of the typical PSI genes. Light affects Pi starvation responses both in local and systemic manners. All these again indicate that the inhibition of PR growth under –Pi condition may be artificial phenotype, encouraging us carefully evaluate the phenotype under illuminated, transparent Petri dishes.

## Data Availability

The datasets presented in this study can be found in online repositories. The names of the repository/repositories and accession number(s) can be found in the article/**Supplementary Material**.
