# Supplementary material for: Physiological and transcriptional analyses of Arabidopsis primary root growth in response to phosphate starvation under light and dark conditions
Source: Front Plant Sci. 2025 Apr 10;16:1557118. doi: 10.3389/fpls.2025.1557118 (PMC12018419; doi:10.3389/fpls.2025.1557118)
Supplement: Supplementary file 1 [file DataSheet1.pdf]

Supplementary Figure 1

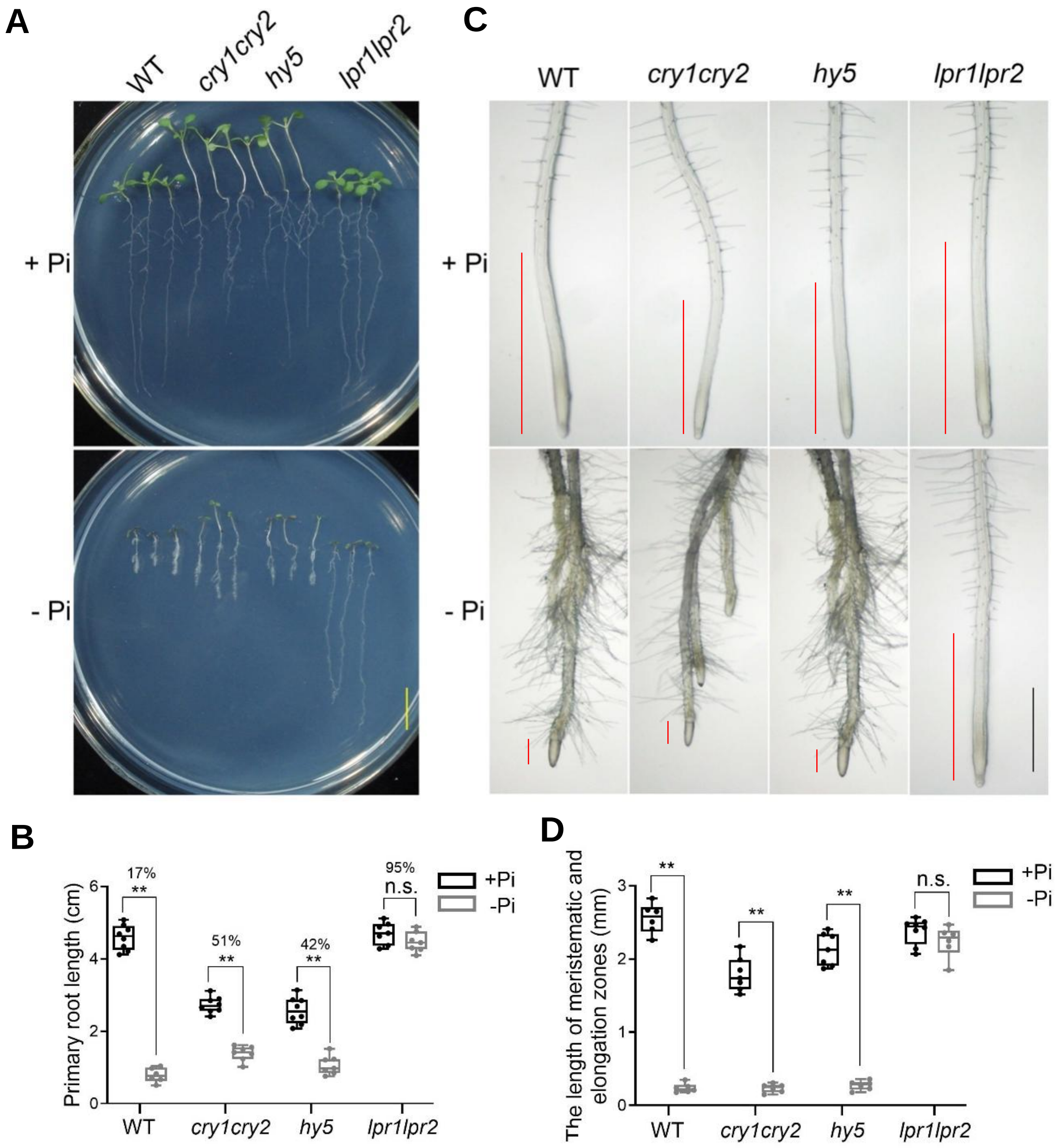

Supplementary Figure 1. The root developmental responses of WT, *cry1cry2*, *hy5*, and *lpr1lpr2* under Pi deficiency in direct germination assay. **(A)** The seeds of the WT, *cry1cry2*, *hy5*, and *lpr1lpr2* were directly germinated on +Pi or –Pi medium and were grown for eight days under light. The scale bar = 1 cm. **(B)** Quantification of the primary root length of the seedlings in (A). The experiments were repeated three times, and the representative results are shown (median ± interquartile; Tukey whiskers; *n* = more than

six seedlings per condition; $**p < 0.01$ in Student's *t*-tests, n.s. represents no significant difference). The percentages represent the relative root lengths calculated by average of primary root length between the –Pi and +Pi conditions. **(C)** Morphologies of the root tips of the seedlings shown in (A). The red lines indicate the meristematic and elongation zones. The scale bar = 1 mm. **(D)** Quantification of the length of meristematic and elongation zones in (C). The experiments were repeated three times, and the representative results are shown (median ± interquartile; Tukey whiskers; *n* = more than six seedlings per condition; $**p < 0.01$ in Student's *t*-tests, n.s. represents no significant difference).

Supplementary Figure 2

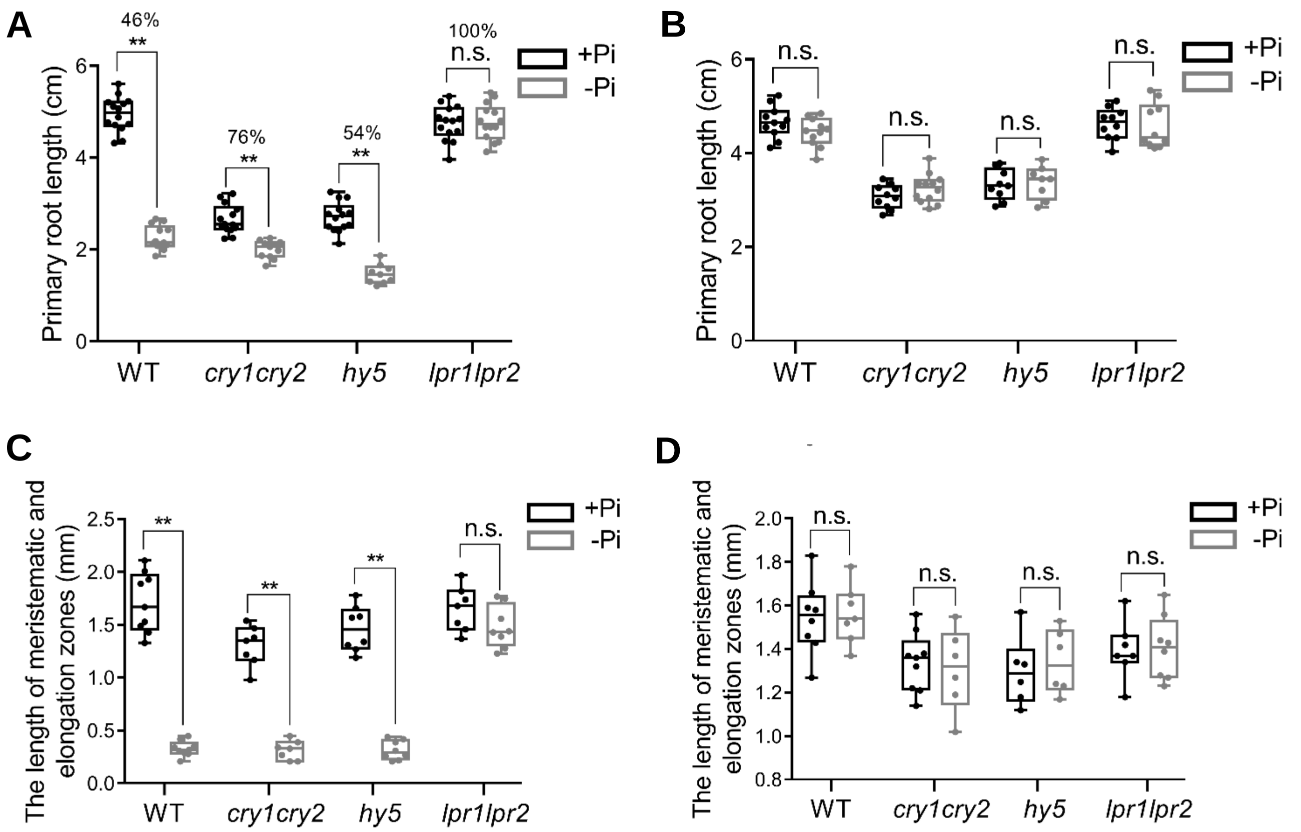

Supplementary Figure 2. Quantification of the primary root length of the seedlings and the length of meristematic and elongation zones in Figure 4A, B. Quantification of the primary root length of the seedlings whose roots were grown under light **(A)** and in darkness **(B)** in Figure 4A. The percentages in (A) represent the relative root lengths calculated by average of primary root length between the –Pi and +Pi conditions. These experiments were repeated three times, and the representative results are shown (median ± interquartile; Tukey whiskers; n = more than eight seedlings per condition; $^{**}p < 0.01$ in Student's t-tests, n.s. represents no significant difference). Quantification of the length of meristematic and elongation zones whose roots were grown under light **(C)** and in darkness **(D)** in Figure 4B. The experiments were repeated three times, and the representative results are shown (median ± interquartile; Tukey whiskers; n = more than six seedlings per condition; $^{**}p < 0.01$ in Student's t-tests, n.s. represents no significant difference).

Supplementary Figure 3

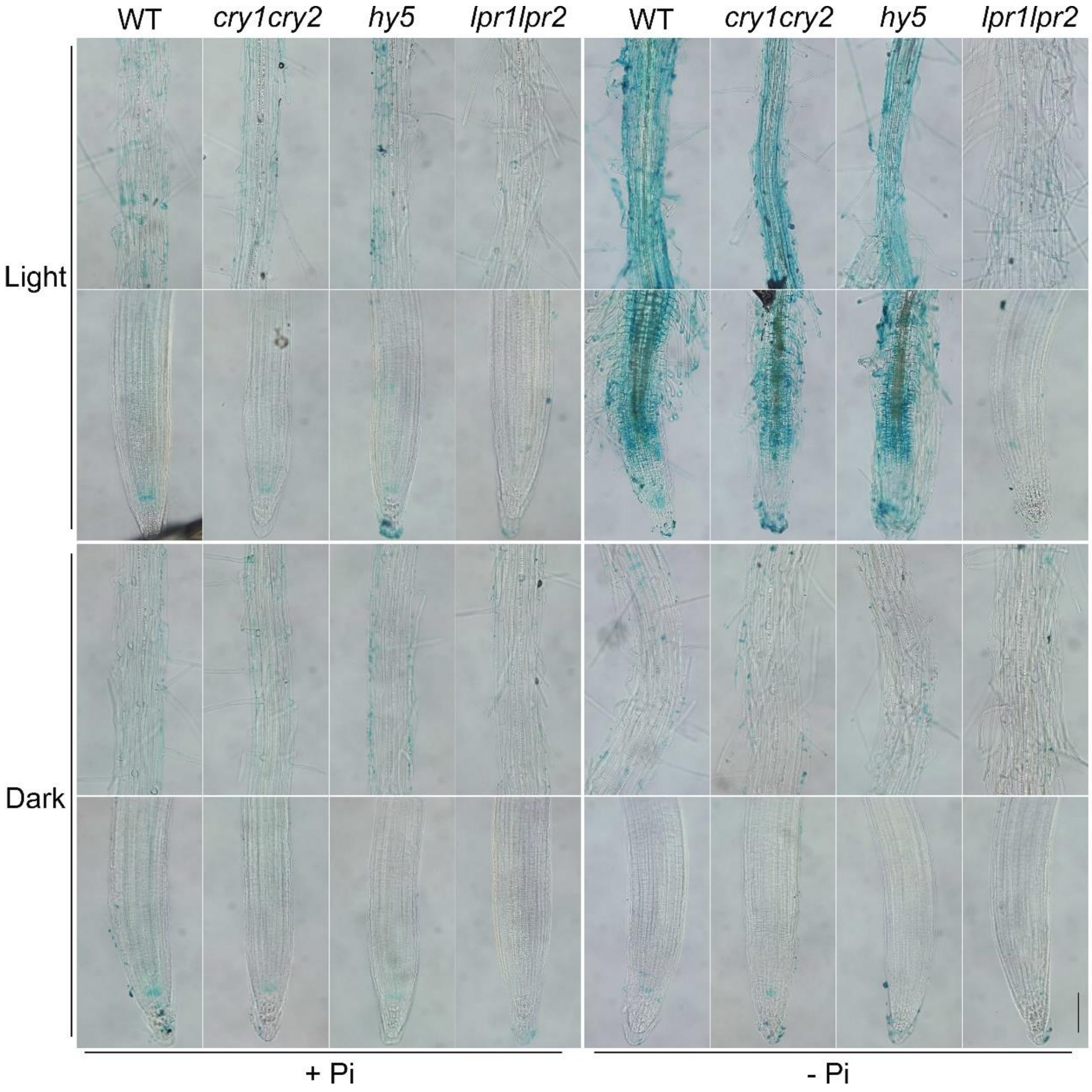

Supplementary Figure 3. Perls staining in the roots of the WT and the various mutants on +Pi and –Pi media under light or in darkness. The 4-day-old +Pi seedlings of the WT, *cry1cry2*, *hy5*, and *lpr1lpr2* were transferred to +Pi or -Pi medium and grown for another four days with shoots grown under light and roots grown under light or in darkness. The root tips were stained using the Perls staining method to reveal the contents of Fe^{3+}. The upper and lower rows in each treatment are photographs of a part of the maturation zone and the root tip, respectively. The scale bar = 100 μm.

Supplementary Figure 4

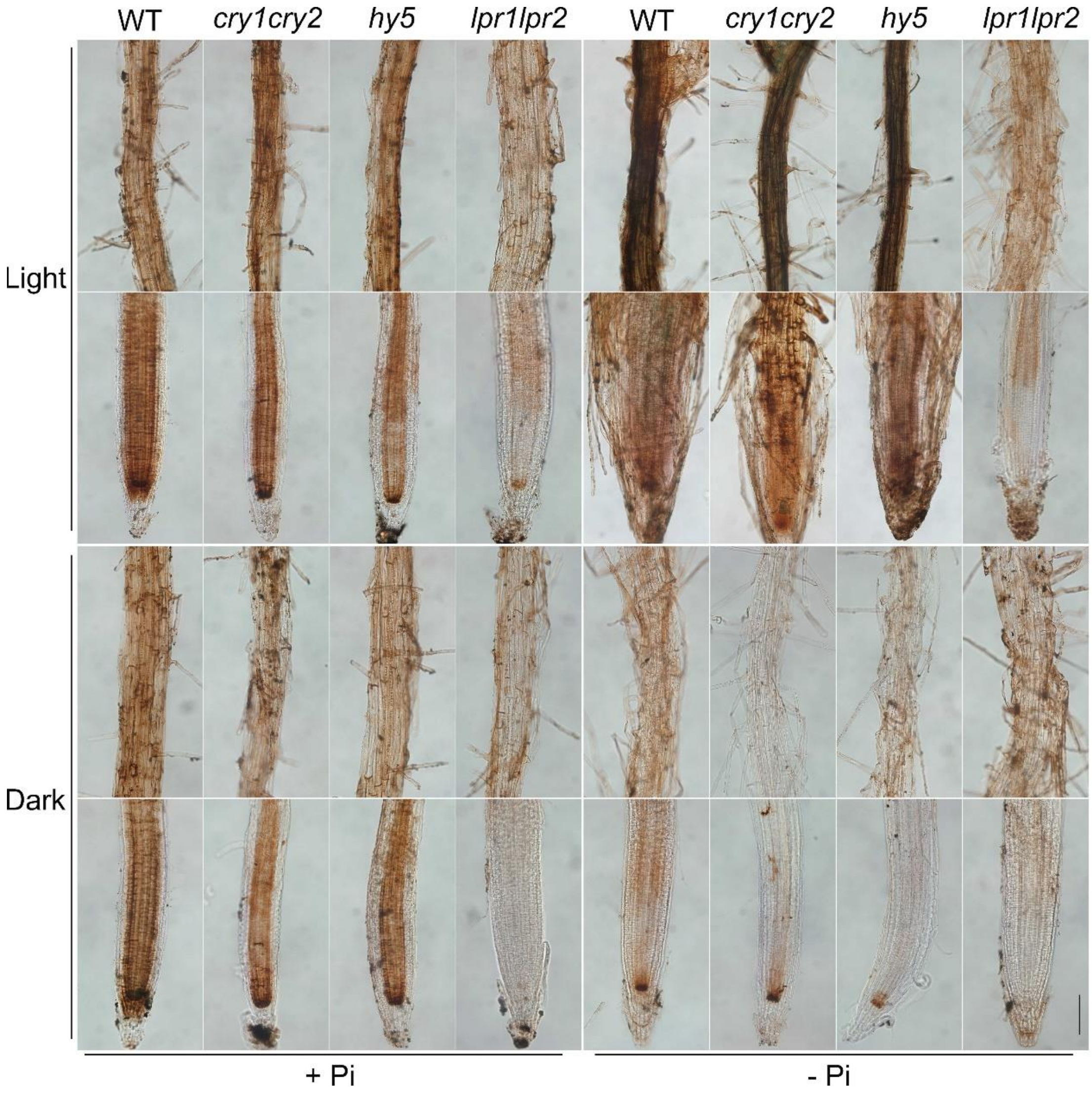

Supplementary Figure 4. Perls/DAB staining in the roots of the WT and the various mutants on +Pi and –Pi media under light or in darkness. The 4-day-old +Pi seedlings of the WT, *cry1cry2*, *hy5*, and *lpr1lpr2* were transferred to +Pi or -Pi medium and grown for another four days with shoots grown under light and roots grown under light or in darkness. The root tips were stained using the Perls/DAB staining method to reveal the contents of total Fe (Fe^{3+} and Fe^{2+} included). The upper and lower rows in each treatment are photographs of a part of the maturation zone and the root tip, respectively. The scale bar = 100 μm.

Supplementary Figure 5

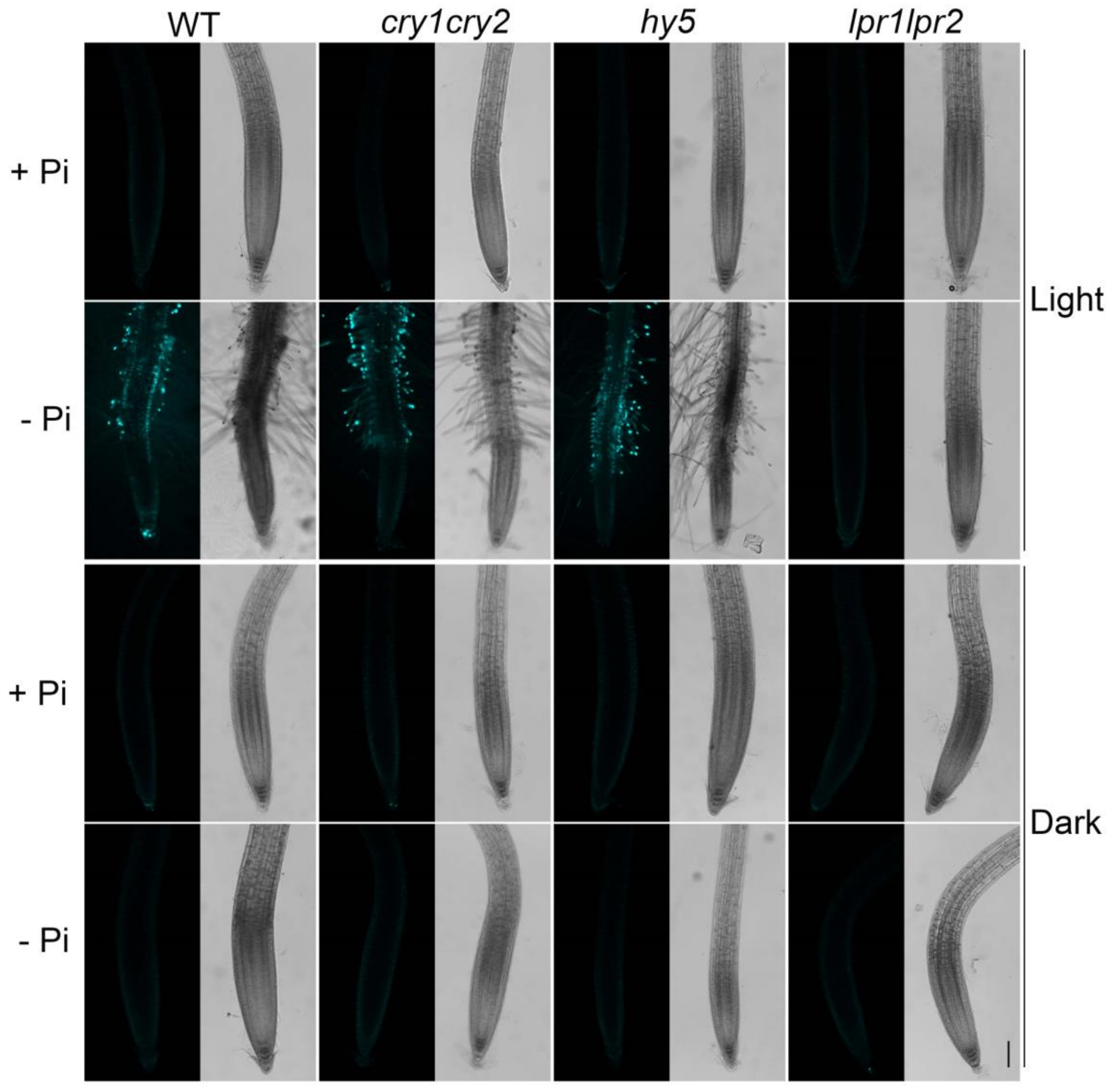

Supplementary Figure 5. The staining of callose in the roots of the WT and the various mutants on +Pi and –Pi media under light or in darkness. The 4-day-old +Pi seedlings of the WT, *cry1cry2*, *hy5*, and *lpr1lpr2* were transferred to +Pi or -Pi medium and grown for another four days with shoots grown under light and roots grown under light or in darkness. Then the callose in the roots was stained by aniline blue. The scale bar = 100 μm.

Supplementary Figure 6

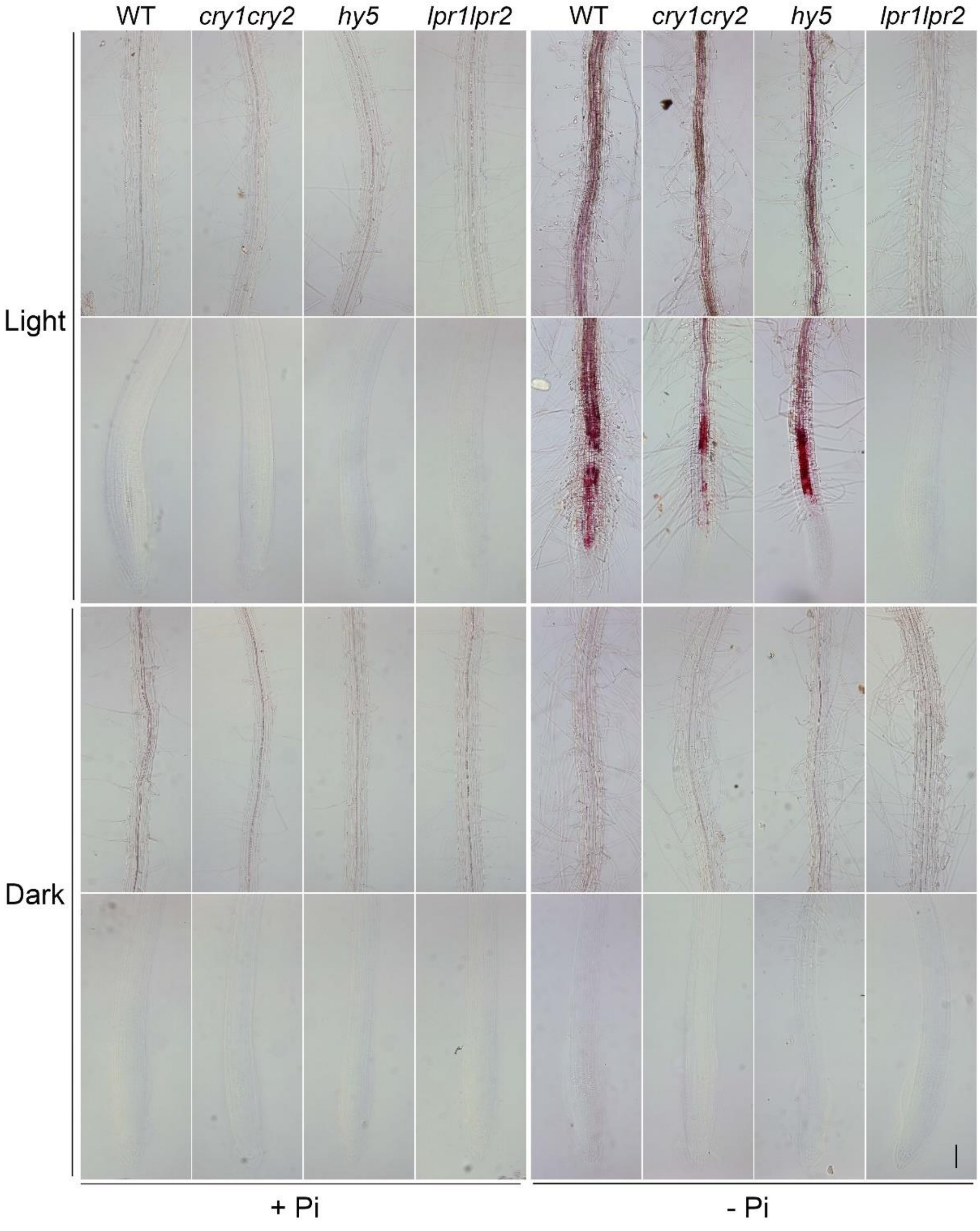

Supplementary Figure 6. The staining of lignin in the roots of the WT and the various mutants on +Pi and –Pi media under light or in darkness. The 4-day-old +Pi seedlings of the WT, *cry1cry2*, *hy5*, and *lpr1lpr2* were transferred to +Pi or -Pi medium and grown for another four days with shoots grown under light and roots grown under light or in darkness. The lignin in the roots was stained by phloroglucinol. The upper and lower

rows in each treatment are photographs of a part of the maturation zone and the root tip, respectively. The scale bar = 100 μm.

Supplementary Figure 7

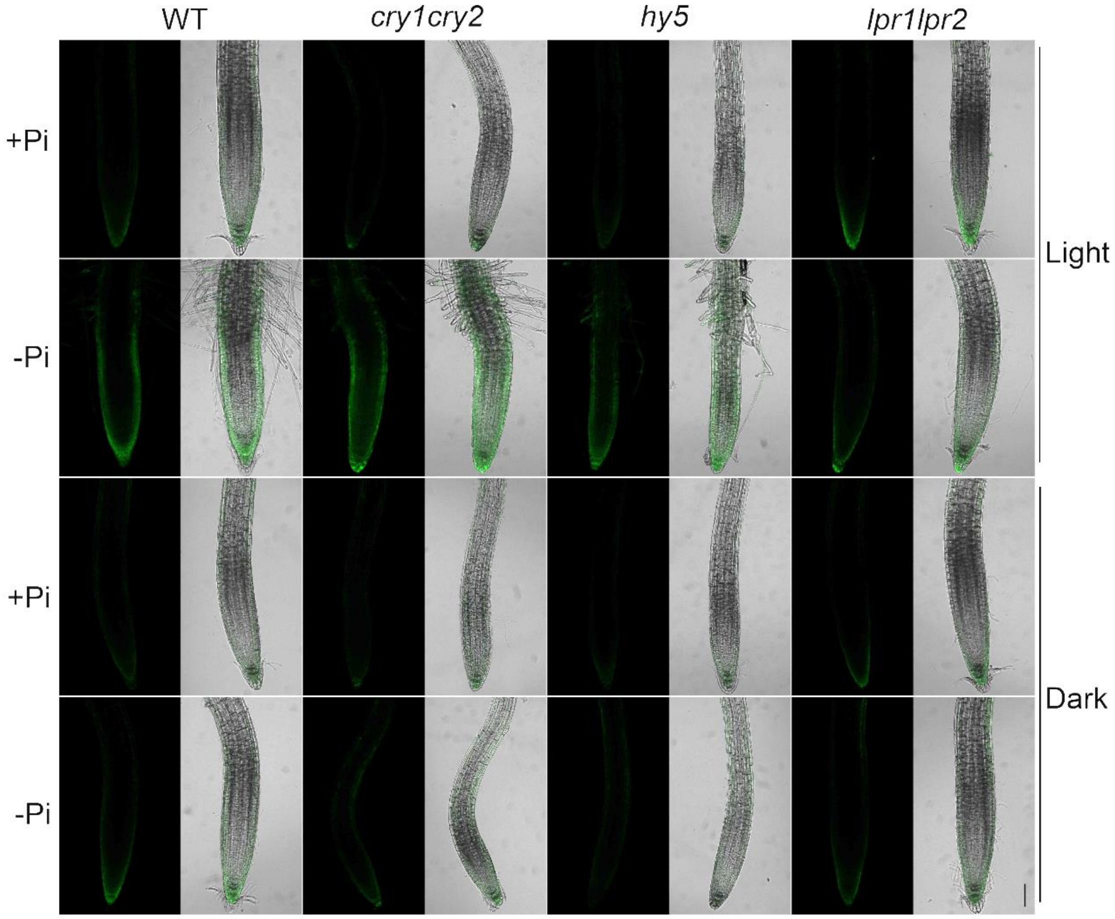

Supplementary Figure 7. The staining of ·OH in the roots of the WT and the various mutants on +Pi and –Pi media under light or in darkness. The 4-day-old +Pi seedlings of the WT, *cry1cry2*, *hy5*, and *lpr1lpr2* were transferred to +Pi or -Pi medium and grown for another two days with shoots grown under light and roots grown under light or in darkness. The ·OH in the roots was stained by HPF. The scale bar = 100 μm.

Supplementary Figure 8

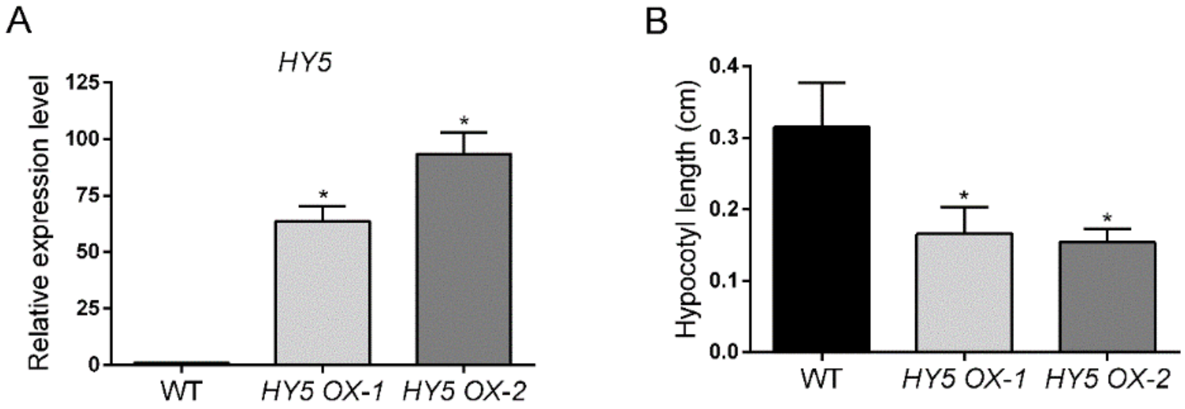

Supplementary Figure 8. Characterizations of the *HY5 OX* lines. **(A)** RT-qPCR analysis of the expression level of *HY5* gene in 8-day-old seedlings of the WT and two *HY5 OX* lines. **(B)** Hypocotyl lengths of 8-day-old seedlings of the WT and two *HY5 OX* lines. Values are means ± SD (n = 12). In **(A)** and **(B)**, *ACTIN2* was used as an internal control. The experiments were repeated three times, and representative results are shown. The values are means ± SD of three samples each with three technical replicates. Asterisks indicate significant differences from the WT (Student's t test, *p < 0.01).

Supplementary Figure 9

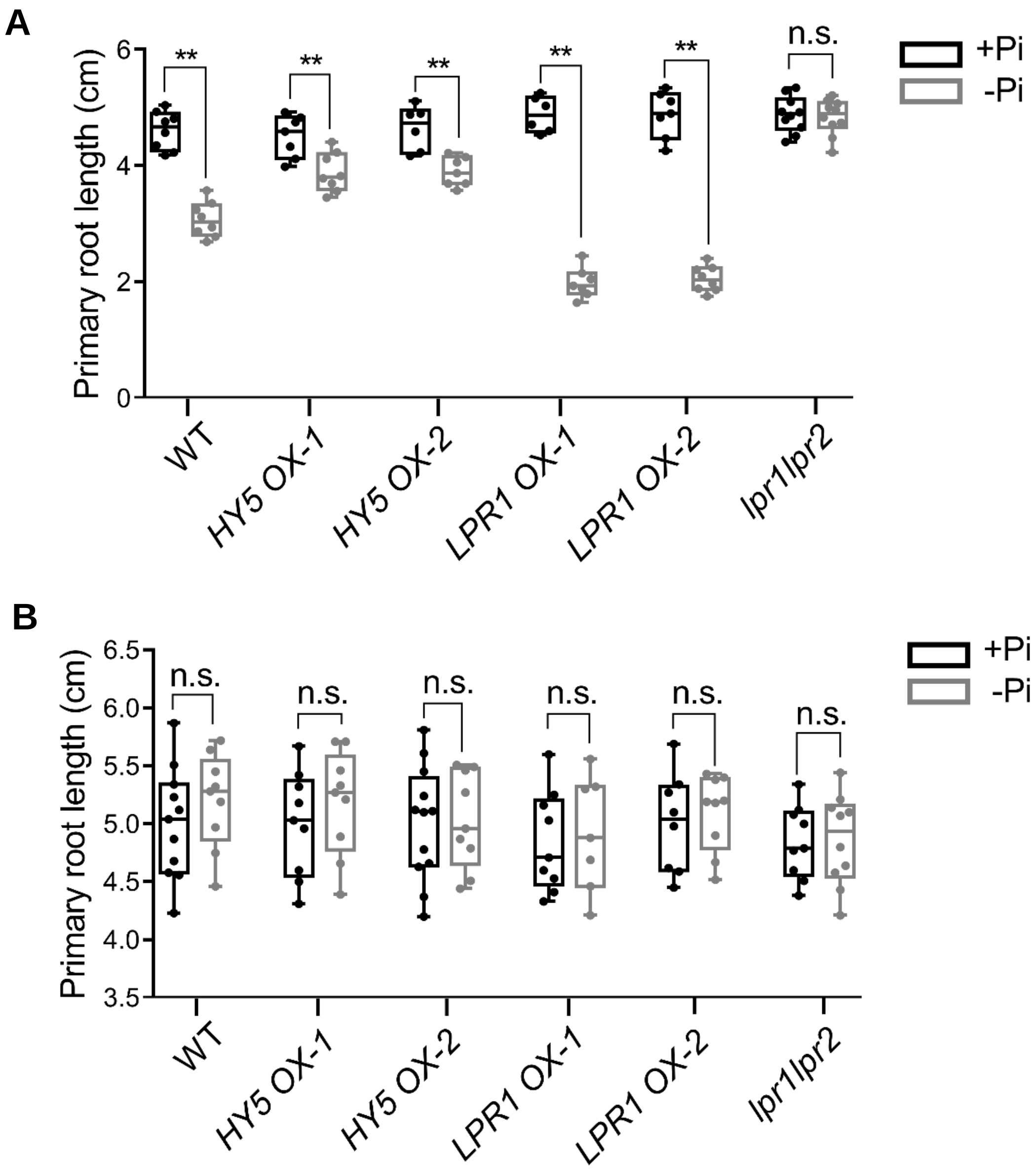

Supplementary Figure 9. Quantification of the primary root length of the seedlings in Figure 4C. Quantification of the primary root length of the seedlings whose roots were grown under light **(A)** and in darkness **(B)** in Figure 4C. These experiments were repeated three times, and the representative results are shown (median ± interquartile; Tukey whiskers; n = more than six seedlings per condition; $^{**}p < 0.01$ in Student's t-tests, n.s. represents no significant difference).

Supplementary Figure 10

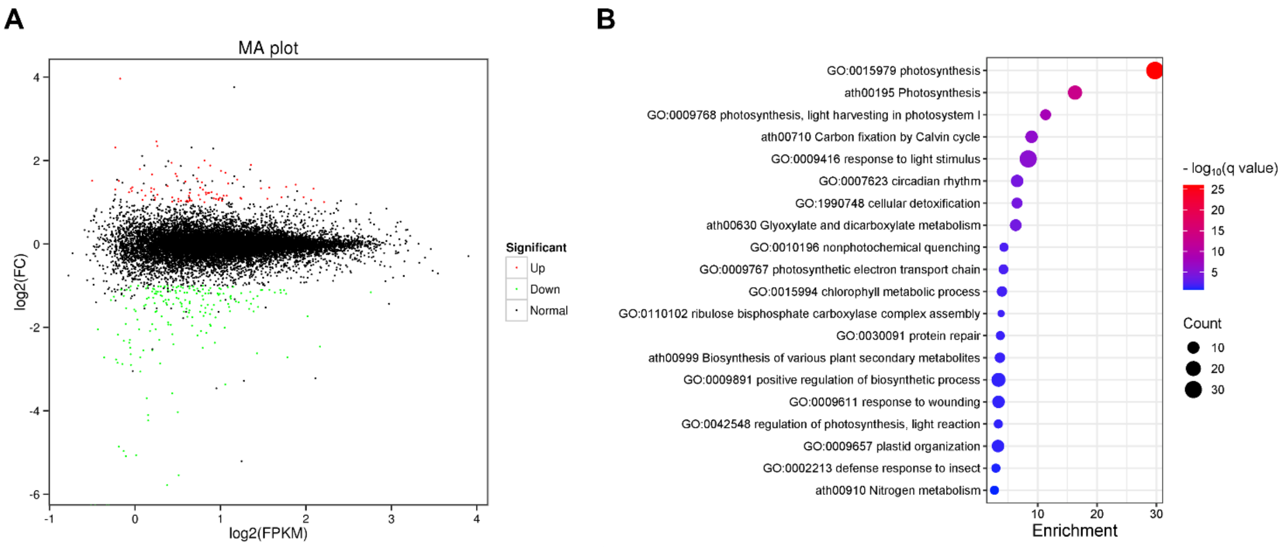

Supplementary Figure 10. MA plot and GO analysis of the DEGs of dark vs. light under +Pi condition. **(A)** MA plot of gene expression level of dark vs. light under +Pi condition. Each dot represents a gene expression level. Red dots mean significantly up-regulated genes; Green dots mean significantly down-regulated genes; black dots mean no significantly changed genes. **(B)** GO analysis of the DEGs of dark vs. light under +Pi condition.

Supplementary Figure 11

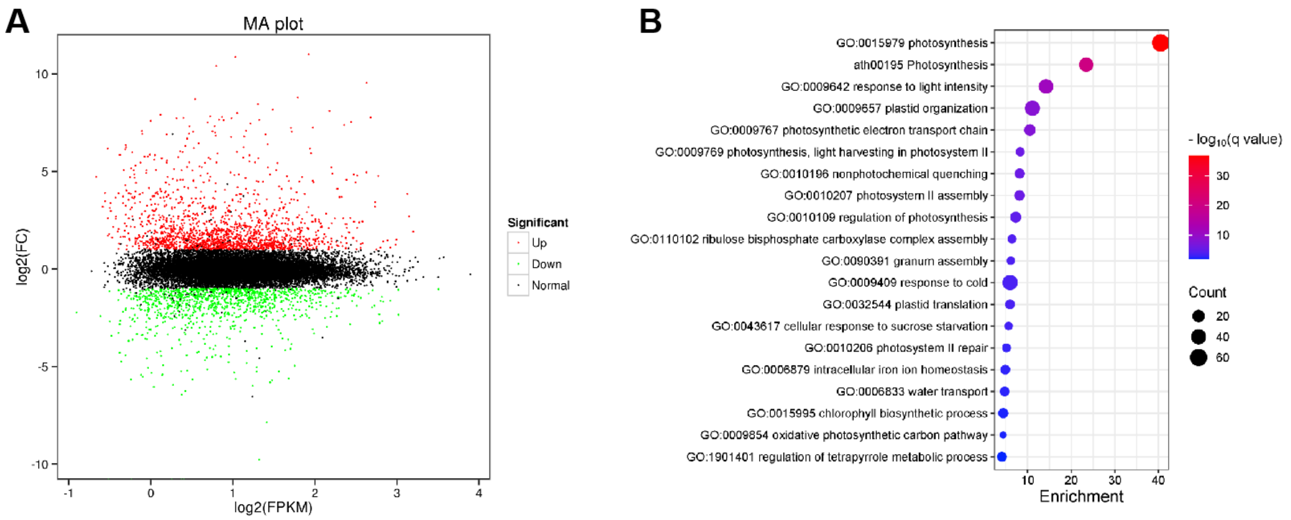

Supplementary Figure 11. MA plot and GO analysis of the light-affected PSS genes. **(A)** MA plot of gene expression level of light-affected PSR genes. Each dot means a gene expression level. Red dots mean significantly up-regulated genes; Green dots mean significantly down-regulated genes; black dots mean no significantly changed genes. **(B)** GO analysis of the light-affected PSS genes.

Supplementary Figure 12

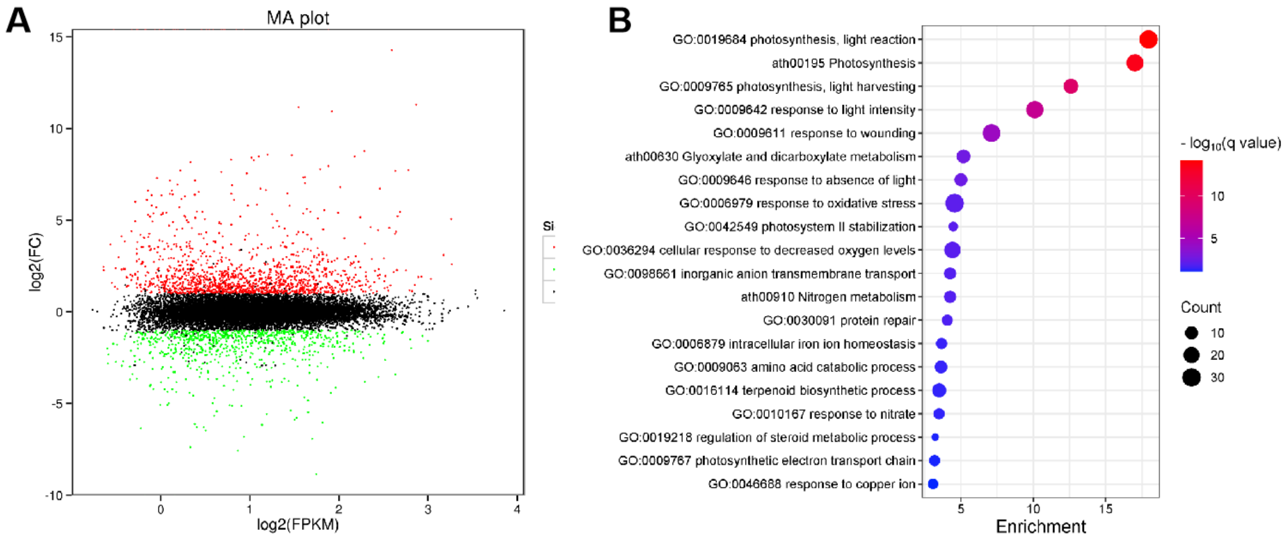

Supplementary Figure 12. MA plot and GO analysis of the dark-affected PSS genes. **(A)** MA plot of gene expression level of dark-affected PSR genes. Each dot means a gene expression level. Red dots mean significantly up-regulated genes; Green dots mean significantly down-regulated genes; black dots mean no significantly changed genes. **(B)** GO analysis of the dark-affected PSS genes.

Supplementary Figure 13

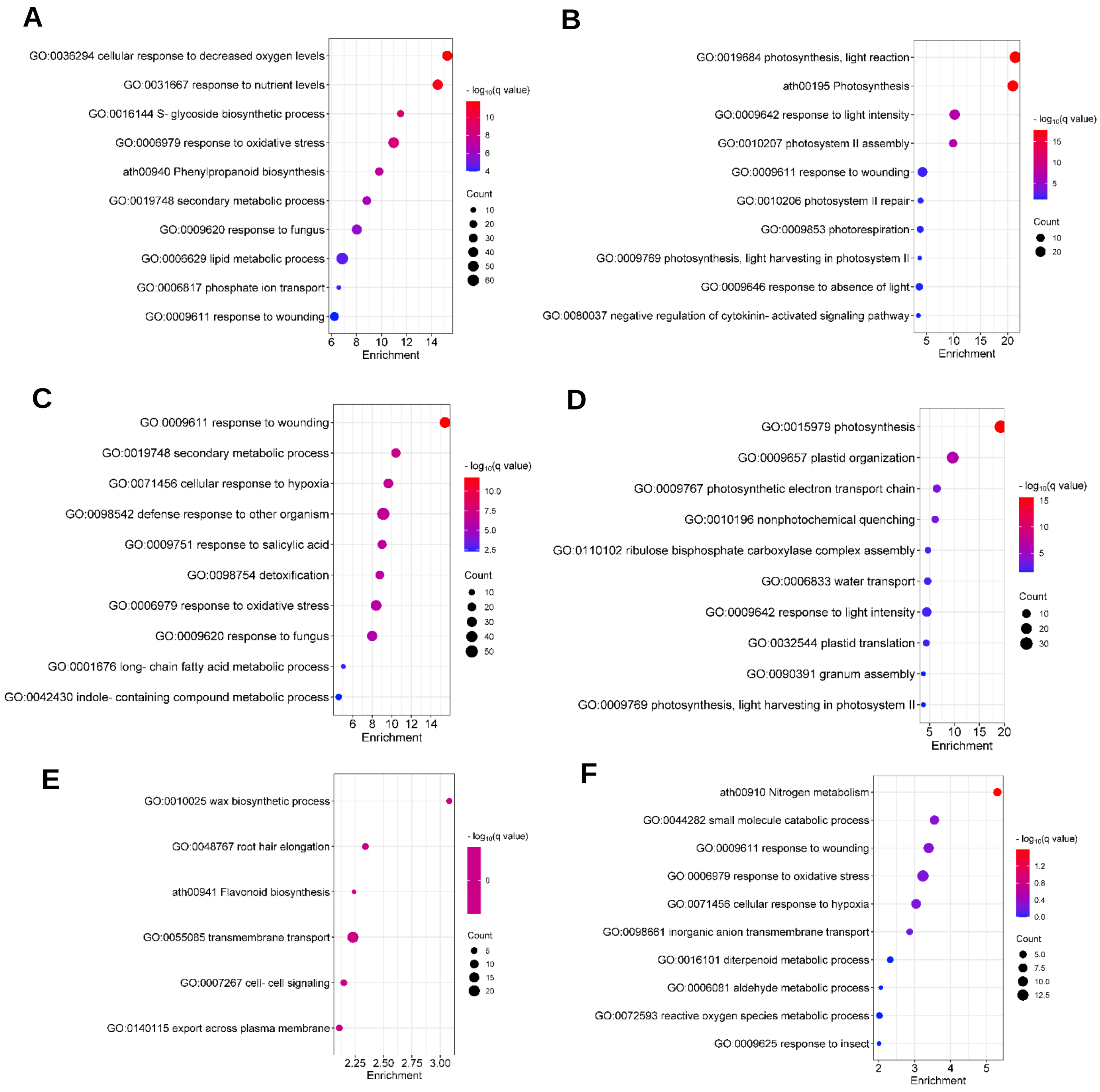

Supplementary Figure 13. The common and specific light- and dark-affected PSR genes. By drawing a Venn diagram, the light- and dark-affected PSR genes were divided into six categories, including GO analyses of the 989 common PSI genes **(A)**, 553 common PSS genes **(B)**, 639 specific light-affected PSI genes **(C)**, 523 specific light-affected PSS genes **(D)**, 413 specific dark-affected PSI genes **(E)**, and 247 specific dark-affected PSS **(F)**.

Supplementary Figure 14

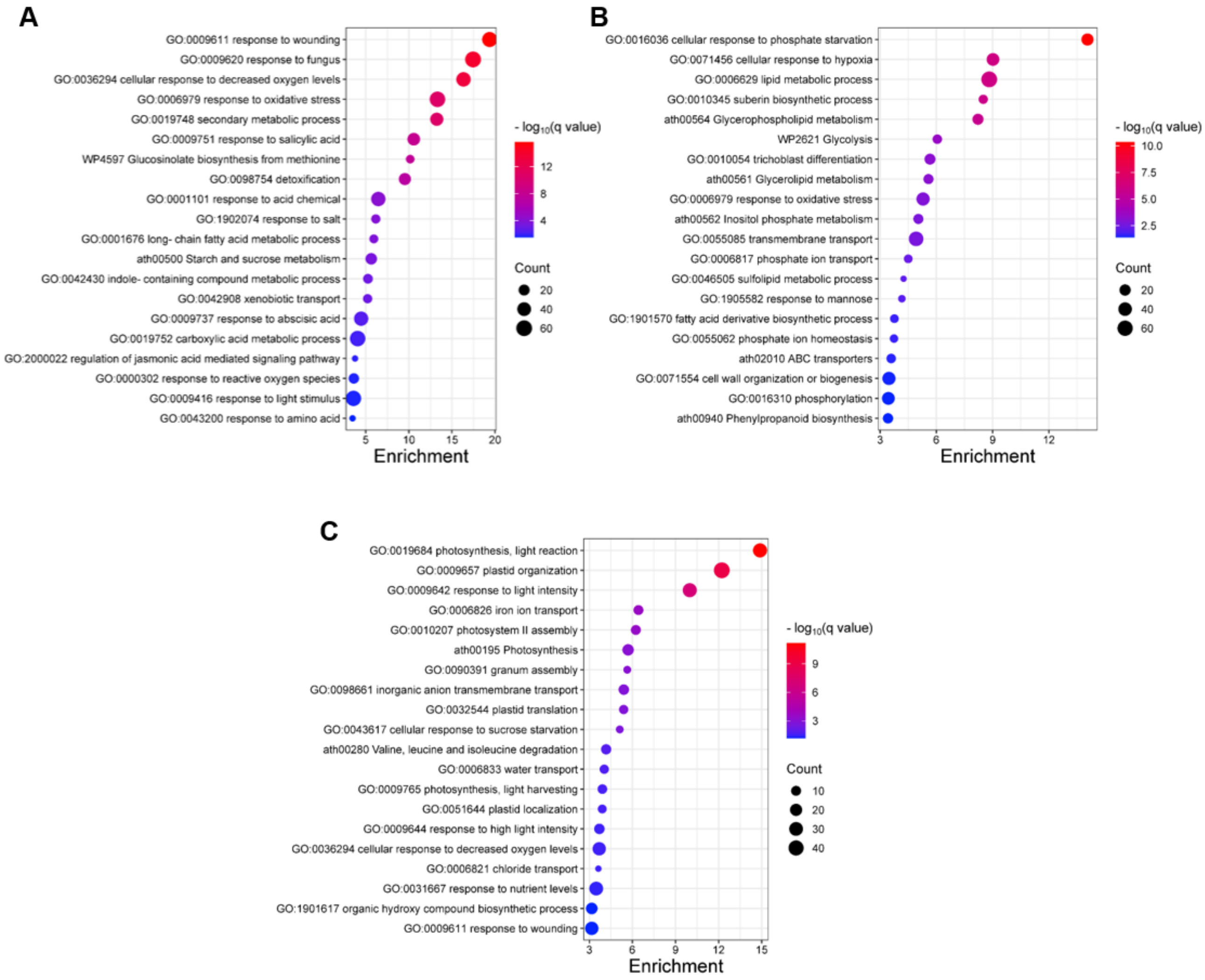

Supplementary Figure 14. The GO analyses of three clusters obtained by hierarchical clustering in the heatmap in Figure 6. GO analyses were conducted for Cluster 1 **(A)**, Cluster 2 **(B)**, and Cluster 3 **(C)**.

Reference

Zhao, S., Ye, Z., and Stanton, R. (2020). Misuse of RPKM or TPM normalization when comparing across samples and sequencing protocols. *RNA* 26, 903-909. doi: 10.1261/rna.074922.120
